# Contribution of proneurotrophin-3 to nerve trauma-induced neuropathic pain through promoting TrkC-mediated increase of CCL2 in primary sensory neurons

**DOI:** 10.1016/j.bbi.2026.106256

**Published:** 2026-01-06

**Authors:** Huijie Shang, Xianglei Meng, Bing Wang, Xiaozhou Feng, Ruining Ma, Huijuan Hu, Alex Bekker, Yuan-Xiang Tao

**Affiliations:** a Department of Anesthesiology, New Jersey Medical School, Rutgers, The State University of New Jersey, Newark NJ07103, USA; b Department of Physiology, Pharmacology & Neuroscience, New Jersey Medical School, Rutgers, The State University of New Jersey, Newark NJ07103, USA; c Department of Cell Biology & Molecular Medicine, New Jersey Medical School, Rutgers, The State University of New Jersey, Newark NJ07103, USA

**Keywords:** Proneurotrophin-3, Tropomyosin receptor kinase C, C-C chemokine ligand 2, Neurotrophin-3, Primary sensory neuron, Nerve trauma, Neuropathic pain

## Abstract

Neuropathic pain induced by nerve trauma remains a substantial and unresolved clinical challenge. Despite ongoing research, therapeutic options for this disorder remain inadequate. Here, we report that neurotrophin-3 (*Nt3*) mRNA and its encoded proneurotrophin-3 (proNT3) protein are upregulated in neurons of the injured dorsal root ganglion (DRG), but not in the spinal cord, following peripheral nerve trauma. Mature neurotrophin-3 (NT3) protein is undetectable in the DRG under both normal and nerve trauma conditions. Genetic blockage of *Nt3* mRNA/proNT3 protein upregulation in the injured DRG attenuates the development and maintenance of nerve trauma-induced neuropathic pain, without impacting acute/basal pain responses or locomotor function. Conversely, mimicking nerve trauma-induced upregulation of DRG *Nt3* mRNA/pro-NT3 produces neuropathic pain-like symptoms. These symptoms are mitigated by intrathecal injection of NT3 protein or by selective knockdown of tropomyosin receptor kinase C (TrkC), but not TrkA or TrkB, in the DRG. Notably, intrathecal injection of NT3 also alleviates nerve trauma-induced neuropathic pain. Mechanistically, upregulated proNT3 contributes to the nerve trauma-induced increases of C-C chemokine ligand 2 (*Ccl2*) mRNA and CCL2 protein through activating TrkC in the injured DRG. Given that CCL2 is a key driver in neuropathic pain genesis and that *Nt3* mRNA co-expresses with *TrkC* and *Ccl2* mRNA in DRG neurons, proNT3 likely participates in nerve trauma-induced neuropathic pain through promoting TrkC-mediated increase of CCL2 in DRG neurons, highlighting a potential therapeutic target for the treatment of this disorder.

## 1. Introduction

Nerve trauma-induced neuropathic pain occurs as a consequence of injury to the peripheral nervous system. This condition affects approximately 7–10 % of the general population worldwide (Cohen and Mao, 2014). Current treatments for this disorder are, unfortunately, inadequate due to poor efficacy and side effects, as exemplified by the opioid crisis (Finnerup et al., 2015; Meyer et al., 2014). There is therefore a pressing need for further understanding pathological mechanisms of nerve trauma-induced neuropathic pain and the development of nonaddictive therapies for this disorder.

C-C chemokine ligand 2 (CCL2), also known as monocyte chemoattractant protein-1 (MCP-1), is a small cytokine that plays a critical role in the peripheral mechanism of neuropathic pain genesis. The levels of CCL2 and its preferred C-C motif receptor 2 (CCR2) are significantly increased in neurons of injured dorsal root ganglion (DRG) following peripheral nerve trauma (Cai et al., 2013; Du et al., 2022; Wang et al., 2010; Wen et al., 2023; White et al., 2009; Zhang et al., 2024). CCL2 directly excites injured small/ medium/large DRG neurons through CCR2 activation by autocrine/paracrine processes (Jung et al., 2008; Sun et al., 2006; Van et al., 2011; White et al., 2005). CCR2 sensitizes nociceptors via transactivation of TRPV1 and Nav1.8 (Belkouch et al.,2011; Jung et al., 2008; Kao et al., 2012; Zhao et al., 2014). Behaviorally, intrathecal CCL2 leads to rapid nociceptive hypersensitivity (Gao et al., 2009; Van et al., 2011). Intrathecal CCR2 antagonists or CCL2 neutralizing antibody alleviate nerve trauma-induced neuropathic pain (Gao et al., 2009; Imai et al., 2013; Van et al., 2011; Zhu et al., 2014). Consistently, CCR2 knockout impairs nerve trauma-induced mechanical allodynia (Abbadie et al., 2003). Thus, CCL2 is a key driver in the induction and maintenance of nerve trauma-induced neuropathic pain. However, the mechanisms underlying the upregulation of CCL2 in injured DRG neurons remain incompletely understood. Elucidating these mechanisms may lead to novel therapeutic strategies for nerve trauma-induced neuropathic pain.

Neurotrophin-3 (NT-3), a member of the neurotrophin family, may be essential for the development, survival, and function of neurons in both central and peripheral nervous systems through its binding to the tropomyosin receptor kinase C (TrkC) or p75 non-tyrosine receptor (NTR), a low-affinity neurotrophin receptor (Bartkowska et al., 2010; Gibon and Barker, 2017). NT-3, like other neurotrophins, is synthesized as a high-molecular-weight precursor known as proneurotrophin-3 (proNT-3; ~32 kDa). This precursor is translated from *Nt3* mRNA and subsequently cleaved by furin or other proprotein convertases to generate C-terminal mature NT-3 form (~14.5 kDa) (Teng et al., 2010; Yano et al., 2009). Both proNT-3 and mature NT-3 are biologically active, but they exert opposing effects (Gibon and Barker, 2017; Teng et al., 2010). The role of NT3 and pro-NT3 in nerve trauma-indued neuropathic pain is still elusive. The expression of *Nt3* mRNA is increased in neurons of injured DRG after peripheral nerve trauma (Kazemi et al., 2017; Wang et al., 2008). Consistently, NT-3-like immunoreactivity is elevated in DRG neurons following resiniferatoxin treatment (Tender et al., 2011) or the removal of adjacent DRGs (Wang et al., 2008). This immunoreactivity should contain both NT3 and proNT3, as NT3 is the C-terminal portion of proNT3 (Teng et al., 2010; Yano et al., 2009). Intrathecal administration of *Nt3* antisense oligonucleotide (ASO), as well as local and systemic delivery of NT-3 antiserum, mitigated nerve trauma-induced mechanical allodynia (Deng et al., 2000; Quintao et al., 2008; White, 2000; Zhou et al., 2000b). The potential contribution of proNT-3 cannot be excluded in these studies. Interestingly, a previous study revealed that intrathecal NT-3 induced mechanical allodynia in normal rats (White, 1998), whereases other studies reported that intrathecal NT3 inhibited nerve trauma-induced thermal hyperalgesia (Wilson-Gerwing et al., 2005) and that NT3 directly delivered into DRG did not affect mechanical nociceptive threshold (Zhou et al., 2000a). Obviously, the expression patterns of NT-3 and proNT-3 in the DRG, as well as their roles in nerve trauma-induced neuropathic pain remain poorly understood. Given that CCL2 has been identified as a potential downstream target of TrkC (Salvador et al., 2014), it remains to be determined whether CCL2 mediates the effects of proNT3/NT3 in nerve trauma–induced neuropathic pain.

In this study, we reported the expression of proNT-3, but not mature NT-3, in the DRG. pro-NT3 was time-dependently upregulated in the injured DRG, but not in spinal cord, in two well-established mouse models of nerve trauma-induced neuropathic pain induced by chronic constriction injury (CCI) of unilateral sciatic nerve or unilateral fourth lumbar spinal nerve ligation (SNL). Furthermore, we demonstrated that this upregulation was both necessary and sufficient for CCI-induced neuropathic pain, acting through promoting TrkC-mediated elevation of CCL2 in the injured DRG.

## 2. Materials and methods

### 2.1. Animal preparations

Adult male and female CD1 mice (7–8 weeks; 25–30 g) were purchased from Charles River Laboratories (Wilmington, MA, USA). The advillin^CreERT2/+^ mice and NT3^f/f^ mice were purchased from Jackson Laboratory (Bar Harbor, ME, USA). Male sensory neuron-specific Advillin^CreERT2/+^ mice were crossed with female NT3^f/f^ mice to obtain inducible conditional NT3 knockdown (NT3 cKD) mice. All mice were kept in the central facility at Rutgers New Jersey Medical School under a standard 12 h light/dark cycle, with water and food pellets available ad libitum. All experimental procedures were approved by the Animal Care and Use Committee at Rutgers New Jersey Medical School and also consistent with the ethical guidelines of the US National Institutes of Health and the International Association for the Study of Pain. The animals were randomly distributed across various groups. The investigators remained blind to the experimental groups throughout the study. All efforts were taken to minimize mouse suffering and to optimize the sample size.

### 2.2. Neuropathic pain models

The mouse models of CCI- or SNL-induced neuropathic pain were conducted as described previously (Du et al., 2022; Li et al., 2020; Pan et al., 2021a; Wang et al., 2023; Zhao et al., 2017). Briefly, mice were anesthetized with 2 % isoflurane. For the CCI model, the surgically exposed unilateral sciatic nerve was partially constricted by placing three loose ligatures (7–0 silk sutures) spaced 1 mm apart, proximal to its trifurcation. For the SNL model, the unilateral L4 spinal nerve was tightly ligated using 7–0 silk suture followed by complete transection distal to the ligation site. Sham mice underwent identical surgical procedure, including nerve exposure, but without ligation and/or transection of the corresponding nerve.

### 2.3. DRG microinjection

DRG microinjection was conducted following the protocols established in our previous studies (Du et al., 2022; Li et al., 2020; Pan et al., 2021a; Wang et al., 2023; Zhao et al., 2017). Briefly, after the mice were anesthetized with 2 % isoflurane, a dorsal midline incision was performed in the lower lumbar region. The articular processes of unilateral L3-L4 vertebrae were carefully excised to expose the corresponding DRGs. A single microinjection of AAV5 (1 μl/DRG; 4–9 × 10^12^ genome copies/ml) or siRNA (1 μl/DRG; 80 μ M; *Nt3*-siRNA (sc-42126) and *TrkA*-siRNA (sc-36727) from Santa Cruz Biotechnology; *TrkB*-siRNA (155697) and *TrkC*-siRNA (143655) from Thermo Fisher Scientific) was performed into the exposed L3/4 DRGs with a glass micropipette connected to a Hamilton syringe under dissection microscopy for 5–10 min. AAV5 was chosen as the delivery vehicle, as AAV5-transduced neuronal populations (mainly in medium and large neurons) in the DRG (Zhao et al., 2013) are similar to the distribution pattern of DRG *Nt3* mRNA-labeled neurons. For optimal delivery and protection of siRNA, TurboFect^™^ *in vivo* transfection reagent (Thermo Fisher Scientific, catalog number: R0531) was used to dissolve siRNA following the manufacturer’s recommended protocol. After microinjection, a 10-minute retention of the glass pipette was performed prior to its withdrawal. Any mice displaying abnormal locomotor activity were excluded from the experiment.

### 2.4. Intrathecal injection

The mice were given intrathecal (i.th.) injections under unanesthetized conditions to avoid the effect of anesthetic on the drugs’ actions as described previously (Du et al., 2022; Li et al., 2020; Pan et al., 2021a; Wang et al., 2023; Zhao et al., 2017). A single i.th. injection was performed by a quick lumbar puncture with a 30 gauge, 0.5 in. needle mated to a 10 ul syringe on the lower back of the mice. Briefly, a sterile needle was inserted into the tissue to one side of the L4 or L5 spinous process so that it slipped into the groove between the spinous and transverse processes. The needle was then moved carefully forward to the intervertebral space. The site of the injection was between L4 and L5. NT3 protein (2 μ g/5μl; Sino Biological, catalog number: 10286-HNAE) or vehicle (5 μl) was injected. All injected mice displayed normal loco motor activity.

### 2.5. Behavioral tests

The evoked pain tests (including mechanical, heat and cold tests), conditional place preference (CPP) test and locomotor function testing were carried out as previously described (Du et al., 2022; Li et al., 2020; Pan et al., 2021a; Wang et al., 2023; Zhao et al., 2017). To minimize stress, mice were allowed to adapt to the test conditions for 1–2 h daily over 2–3 days before behavioral tests.

Paw withdrawal frequencies in response to mechanical stimuli were measured with two calibrated von Frey filaments (0.07 and 0.4 g, Stoelting Co., Wood Dale, IL, USA). Briefly, mice were placed in Plexiglas chambers on an elevated mesh screen for 30 min. The mid-plantar area of each hind paw was stimulated 10 times with von Frey filaments at 5-minute intervals. A quick withdrawal of the hind paw was scored as a positive response, and the percentage withdrawal frequency was calculated as the number positive responses within 10 trials.

Paw withdrawal latencies in response to noxious heat stimuli were examined. Briefly, mice were individually placed in a Plexiglas chamber on a glass platform. A focused light beam, emitted from the Model 336 Analgesia Meter (IITC Inc. Life Science Instruments. Woodland Hills, CA, USA), was directed at the central area of the plantar surface of each hind paw. A quick lift of the hind paw was regarded as a signal to turn off the light. The length of time between the start and the stop of the light beam was defined as the paw withdrawal latency. For each side, three trials at 5-min intervals were carried out. A cutoff time of 20 s was used to avoid tissue damage to the hind paw.

Paw withdrawal latencies in response to noxious cold stimuli were examined. In brief, mice were placed a Plexiglas chamber on the cold aluminum plate (−1 to 0 °C), the temperature of which was monitored continuously by a thermometer. Paw withdrawal latency was defined as the length of time between placement and the first sign of the mouse jumping and/or flinching. Each trial was conducted three times with 10-minute intervals. A cut-off time of 20 s was set to prevent tissue injury.

The CPP test was performed following previously established protocols with minor modifications (Du et al., 2022; Li et al., 2020; Pan et al., 2021a; Wang et al., 2023; Zhao et al., 2017). Briefly, the apparatus consisted of two Plexiglas chambers connected by a central door (Med Associates Inc., Fairfax, VT, USA). One chamber featured a rough floor and horizontal black-and-white striped walls, whereas the other had a smooth floor with vertical black-and-white stripes. Mouse movement and time allocation in each chamber were automatically tracked by photobeam sensors mounted along the chamber walls and recorded using MED-PC IV CPP software. Mice were initially allowed to explore both chambers freely for 30 min during the preconditioning phase to acclimate to the environment. At the end of this session, the time spent in each chamber was recorded over a 15-minute period to evaluate any inherent side preference. Mice that spent more than 80 % or less than 20 % of the total time in a single chamber during preconditioning were excluded from subsequent testing. Conditioning was conducted over the following three days with the internal door between chambers kept closed. The mice received an i.th. injection of saline (5 μl) and were confined to one designated chamber for 15 min. Six hours later, a second i.th. injection of lidocaine (0.8 % in 5 μl saline) was administered and paired with the opposite chamber for another 15-minute session in the afternoon. The sequence of saline and lidocaine administration was alternated daily. On the final test day, mice were given unrestricted access to both chambers with the door open. Their movements and the time spent in each chamber were recorded over a 15-minute period to evaluate chamber preference. A difference score was then calculated by subtracting the time spent in the lidocaine-paired chamber during the preconditioning phase from that during the test phase.

Locomotor function was examined using the following three reflexes. For the placing reflex, the placed positions of the hind limbs were slightly lower than those of the forelimbs, and the dorsal surfaces of the hind paws were brought into contact with the edge of a table. Whether the hind paws were placed on the table surface reflexively was recorded. For the grasping reflex, after the mouse was placed on a wire grid, whether the hind paws grasped the wire on contact was recorded. For the righting reflex, when the mouse was placed on its back on a flat surface, whether it immediately assumed the normal upright position was recorded. Each trial was repeated 5 times at 5-min intervals and the scores for each reflex were recorded based on counts of each normal reflex.

### 2.6. Quantitative real-time reverse transcription polymerase chain reaction (RT-PCR) assay

RNA extraction and quantitative real-time RT-PCR assay were carried out according to our published protocol with minor modifications (Du et al., 2022; Li et al., 2020; Pan et al., 2021a; Wang et al., 2023; Zhao et al., 2017). In brief, two DRGs were pooled to obtain sufficient RNA. Total RNA was extracted using the miRNeasy Kit (Qiagen, Valencia, CA, USA) in accordance with the manufacturer’s protocol. cDNA synthesis was carried out using Thermo Script Reverse Transcriptase (Invitrogen, Thermo Fisher Scientific, Waltham, MA, USA) with oligo(dT) primers. The template (4 μl) was amplified in a Bio-Rad CFX96 real-time PCR system with the primers listed in Supplementary Table 1. PCR reactions were performed in triplicate for each sample using a 20 μL total volume. Each reaction contained 250 nM of both forward and reverse primers, 10 μL Advanced Universal SYBR Green Supermix (Bio-Rad Laboratories), and 20 ng of cDNA template. The amplification protocol involved 40 cycles of denaturation at 95 °C for 30 s, annealing at 60 °C for 30 s, and extension at 72 °C for 30 s. All PCR results were normalized using *Tuba1α* as the internal control. Relative mRNA expression levels were determined with the ΔCt method (2^^−ΔΔCt^).

### 2.7. Western blotting assay

Protein extraction and Western blotting were carried out according to our published protocols (Du et al., 2022; Li et al., 2020; Pan et al., 2021a; Wang et al., 2023; Zhao et al., 2017). Briefly, four DRGs were pooled to obtain sufficient protein. DRGs or spinal cord were homogenized with the lysis buffer containing proteinase inhibitor and phosphatase inhibitor. Following centrifugation at 1,000 × g for 15 min at 4 °C, the supernatant (membrane/cytosolic fractions) was collected. Protein concentrations were measured using the Bio-Rad protein assay (Bio-Rad Laboratories, Hercules, CA, USA). Twenty μg per sample or 0.05 μg recombinant human NT3 protein (SinoBiological, Wayne, PA) were then heated at 99 °C for 5 min and subsequently loaded onto an SDS-polyacrylamide gel consisting of a 4–15 % stacking gel and a 7.5 % separating gel (Bio-Rad Laboratories). Proteins were subsequently transferred onto a polyvinylidene difluoride (PVDF) membrane (Bio-Rad Laboratories) via electrophoresis. The membrane was then blocked for 2 h at room temperature with 5 % non-fat milk dissolved in Tris-buffered saline with 0.1 % Tween-20, followed by overnight incubation at 4 °C with the primary antibodies. These antibodies included rabbit anti-NT3 (1:250; Alomone Labs, catalog number: ANT-003; Jerusalem, Israel), rabbit anti-MCP-1 (1:1000, catalog number: ab315478; Abcam; Waltham, MA), rabbit anti-TrkA (1:1000, catalog number: 2505; Cell Signaling Technology), rabbit anti-TrkB (1:1000, catalog number: GTX133722; GeneTex), rabbit anti-TrkC (1:400, catalog number: ANT-020; Alomone), rabbit anti-ERK1/2 (1:1000, catalog number: 4695; Cell Signaling Technology), rabbit anti-phosphorylated ERK1/2 (p-ERK1/2; 1:1000; catalog number: 4370; Cell Signaling Technology), mouse anti-GFAP (1:1000, catalog number: 3670; Cell Signaling Technology), rabbit anti-IBA1 (1:1000, catalog number: 17198; Cell Signaling Technology), rabbit anti-GAPDH (1:1000, catalog number: G9545; Sigma-Aldrich), or rabbit anti-α-tubulin (1:1000, catalog number: 2144; Cell Signaling Technology). The antibody against NT3 recognized both NT3 and proNT3, which were differentiated based on their distinct molecular weights (proNT3: 32 kDa. NT3: 14.5 kDa). Protein signals were visualized using horseradish peroxidase (HRP) conjugated secondary antibodies anti-mouse or rabbit (1:3000; Jackson ImmunoResearch Laboratories, West Grove, PA, USA). Protein detection was performed with Clarity Western ECL Substrate (Bio-Rad), which includes peroxide solution and luminol/enhancer reagents. Chemiluminescent signals were captured using the ChemiDoc XRS imaging system and analyzed with Image Lab software (Bio-Rad Laboratories). The intensities of the band were analyzed through densitometry with Image Lab software (Bio-Rad). All bands were normalized to α-tubulin or GAPDH.

### 2.8. In situ hybridization histochemistry and co-immunohistochemistry

After mice were deeply anesthetized with isoflurane, they were transcardially perfused with 25–30 ml of 0.1 M phosphate-buffered saline (PBS, pH 7.4), followed by 30–50 ml of 4 % paraformaldehyde prepared in 0.1 M PBS. Following perfusion, L3/4 DRGs were collected, then post-fixed in the same paraformaldehyde solution at 4 °C for 4–6 h and cryoprotected in 30 % sucrose overnight. 15-μm transverse sections were cut on a cryostat. Every third section was collected from each DRG. *In situ* hybridization histochemistry (ISHH) was carried out using a protocol tailored to the IsHyb In Situ Hybridization (ISH) Kit (K2191050; Miramar Beach, FL) with minor modification (Pan et al., 2021b). A probe specific to *Nt3* mRNA (*Nt3* probe; Supplementary Table 1) was prepared by *in vitro* transcription and labeled with digoxigenin-dUTP according to the manufacturer’s instructions (Roche Diagnostics, Indianapolis, IN). The sections were first treated with proteinase K (25 μg/mL; Roche, catalog number: 3115828001) at 37 °C for 10 min and then subjected to prehybridization using an ISH kit (Bio-Chain Institute Inc, CA, USA) for 1.5 h at 62 °C. The sections were hybridized over two nights at 62 °C with digoxigenin-dUTP–labeled *Nt3* probe at a concentration of 8 ng/μl. The sections were then washed sequentially with 2 × SSC and 1.5 × SSC for 20 min each at 62 °C, followed by two washes with 0.2 × SSC for 20 min at 37 °C. After being blocked for 1 h at room temperature in 0.01 M PBS with 0.3 % Triton X-100 and 4 % goat serum, the sections were incubated with an AP-conjugated anti-digoxigenin antibody along with chicken anti-β-tubulin III (1:1,000, catalog number: AB9354; MilliporeSigma, MA, USA) or mouse anti-glutamine synthetase (1:1,000, MAB302; MilliporeSigma, MA,USA) overnight at 4 °C. The sections were then incubated at room temperature for 1 h with a species-appropriate fluorescence-conjugated secondary antibody (Jackson ImmunoResearch, West Grove, PA, USA) and Fast Red. The immunofluorescent images were captured under a Leica DMI4000 fluorescence microscope (Leica) with a DFC365 FX camera (Leica). Number of the neurons (with nucleus) double-labeled by *Nt3* mRNA and each marker and number of the neurons (with nucleus) single labeled by each marker in each section were counted. At least 3–4 sections per DRG were examined. Average percentage of *Nt3* mRNA positive neurons per section within each marker was calculated.

### 2.9. Single-cell RT-PCR assay

The freshly cultured DRG neurons from adult mice (≥ 4 weeks) were prepared as previously described (Du et al., 2022; Li et al., 2020; Pan et al., 2021a; Wang et al., 2023; Zhao et al., 2017). Four hours after seeding, a single living DRG neuron (large: > 35 μm; medium: 25–35 μm; small: < 25 μm) was collected under an inverted microscope using a micromanipulator and microinjector into a PCR tube containing 6–8 μl of cell lysis buffer (Signosis, Sunnyvale, CA). After centrifugation, the lysis solution was aliquoted into distinct PCR tubes for different genes. The RT-PCR procedure was carried out with the Single-Cell RT-PCR Assay Kit according to the instructions provided by the manufacturer (Signosis). The nest-PCR primers used were listed in Supplementary Table 1. The PCR products were analyzed on ethidium bromide-stained 2 % agarose gels.

### 2.10. Plasmid construction and virus production

Full-length *Nt3* mRNA sequences extracted from mouse DRG were reverse-transcribed using the SuperScript IV One-Step RT-qPCR System with the Platinum Taq High Fidelity Kit (Invitrogen/Thermo Fisher Scientific) and amplified by PCR with gene-specific primers listed in Supplementary Table 1. The amplified fragment was inserted into the proviral plasmid at the XhoI and NotI restriction sites. Recombinant clones were verified by DNA sequencing. The resulting vectors expressed the genes under the control of the cytomegalovirus (CMV) promoter.

For AAV5 packaging, HEK-293 cells were transduced with the proviral plasmid expressing either *Nt3* mRNA or GFP using the PEI transduction method with pHelper. Three days post-transduction, cells were harvested, and AAV5 particles were purified using the AAVpro Purification Kit (Takara, Mountain View, CA). Viral titer was determined using the AAV Real-Time PCR Titration Kit (Takara).

### 2.11. Statistical analysis

The mice were randomly assigned to different treatment groups. All results are expressed as mean ± standard error of the mean (SEM). Although data were assumed to follow a normal distribution, this was not formally tested. Statistical analyses were performed with two-tailed unpaired Student’s t-tests or one-/two-/three-way ANOVA, as appropriate. When ANOVA indicated significance, post hoc comparisons were conducted with Tukey’s test. *P* < 0.05 was considered indicative of statistical significance.

## 3. Results

### 3.1. Peripheral nerve trauma upregulates the expression of Nt3 mRNA and proNT3 protein in injured DRG neurons

**Fig. 1.**
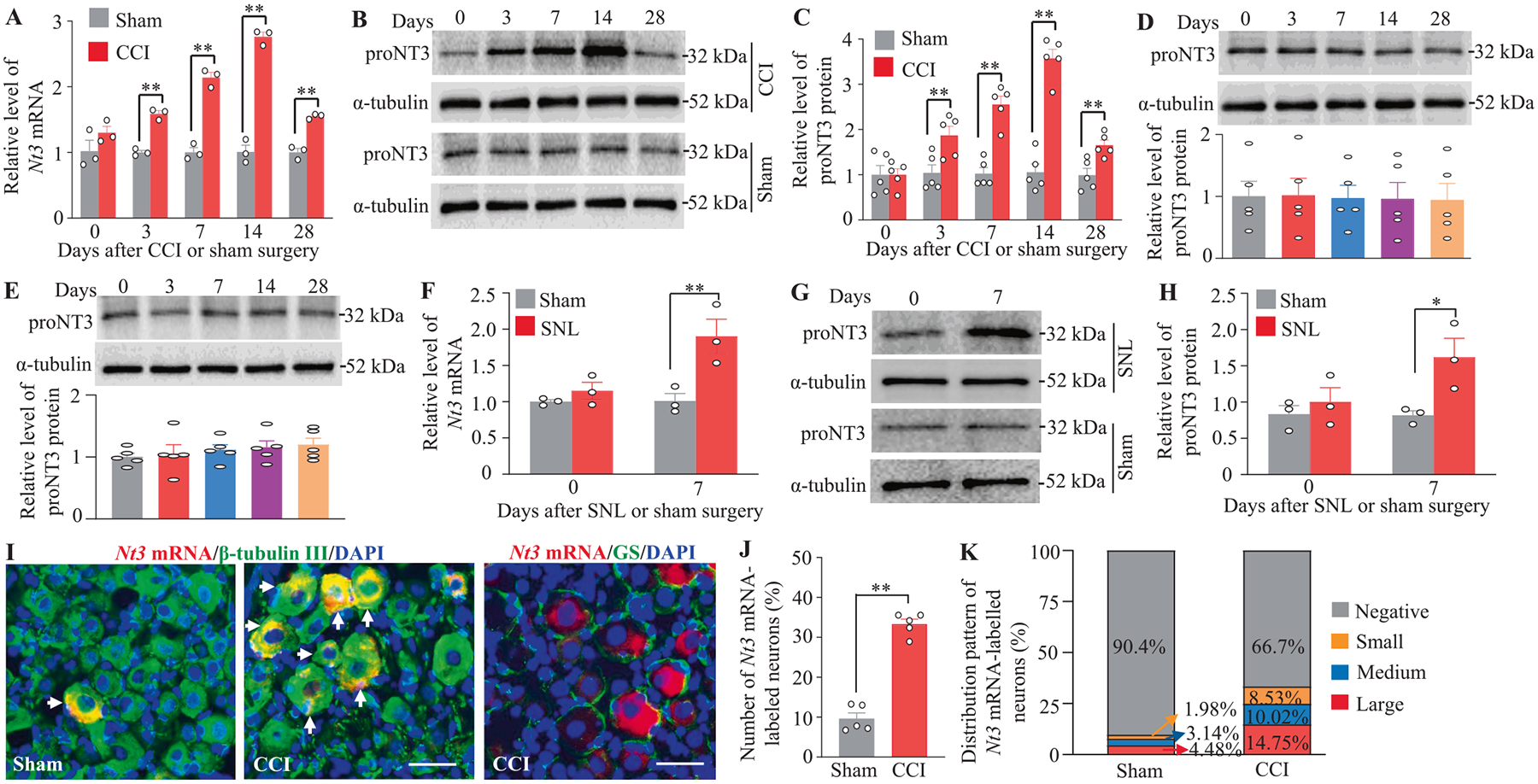
Expression of *Nt3* mRNA and proNT3 protein is upregulated in the injured DRG after peripheral nerve trauma. (A and B) Expression of *Nt3* mRNA (A) and proNT3 protein (B and C) in the ipsilateral L3/4 DRGs on days 0, 3, 7, 14 and 28 after CCI or sham surgery. n = 3–5 repeats (6–10 mice)/group/time point. **P <0.01 by two-way ANOVA followed by post hoc Tukey test. (D and E) Expression of proNT3 protein in the contralateral L3/4 DRGs (D) and ipsilateral L3/4 spinal cord dorsal horn (E) on days 0, 3, 7, 14 and 28 after CCI surgery. n = 5 repeats (10 mice)/group/time point. One-way ANOVA followed by post hoc Tukey test. (F-H) Expression of *Nt3* mRNA (F) and proNT3 protein (G and H) in the ipsilateral L4 DRG on days 0 and 7 after SNL or sham surgery. n = 3 repeats (6–12 mice)/group/time point. *P < 0.05, **P < 0.01 by two-way ANOVA followed by post hoc Tukey test. (I) Representative images of ISHH for *Nt3* mRNA (red) and immunohistochemistry for β-tubulin III (green) or glutamine synthetase (GS; green) in the ipsilateral L4 DRG on day 7 after CCI or sham surgery. Cellular nuclei were labelled by 4′, 6-diamidino-2- phenylindole (DAPI; blue). Arrows: double-labeling neurons. Scale bars: 40 μm. (J and K) Statistical summary for number of *Nt3* mRNA–labeled neurons (J) and cellular distribution of *Nt3* mRNA–labelled neuronal soma (K) in the ipsilateral L3/4 DRGs on day 7 after CCI or sham surgery. n = 5 mice/group. **P < 0.01 by the 2-tailed unpaired Student’s *t* test.

To clarify the role of NT3 and proNT3 in nerve injury-induced neuropathic pain, we first carried out quantitative real-time RT-PCR assay using the specific primers for *Nt3* mRNA and Western blot assay using a specific antibody against both NT3 and proNT3 (Supplementary Fig. 1A) (Shen et al., 2013; Yano et al., 2009) and examined their abundances in DRG and spinal cord, two major pain regions, following the unilateral CCI. The expression of *Nt3* mRNA and proNT3 protein was time-dependently upregulated in the ipsilateral L3/4 DRGs after CCI (Fig. 1A–C). NT3 protein was undetectable in the DRG of naïve mice and CCI/SNL mice (Supplementary Fig. 1B–C). The level of *Nt3* mRNA in the ipsilateral L3/4 DRGs was elevated by 1.6 (*P* < 0.01)-, 2.1 (*P* < 0.01)-, 2.7 (*P* < 0.01)-, and 1.6 (*P* < 0.01)-fold on days 3, 7, 14, and 28, respectively, after CCI as compared to the corresponding sham mice (Fig. 1A). Consistently, the amount of proNT3 protein in the L3/4 DRGs was increased by 1.8 (*P* < 0.01)-, 2.5 (*P* < 0.01)-, 3.4 (*P* < 0.01)-, and 1.7 (*P* < 0.01)-fold on days 3, 7, 14, and 28, respectively, after CCI as compared to the corresponding sham mice (Fig. 1B–C). As expected, sham surgery did not alter the basal expression of *Nt3* mRNA and proNT3 protein in the ipsilateral L3/4 DRGs during the observation period (Fig. 1A–C). CCI did not change the basal expression of proNT3 protein in the contralateral L3/4 DRGs and ipsilateral L3/4 spinal cord dorsal horn on days 3, 7, 14 and 28 post-surgery (Fig. 1D–E). Similarly, the levels of *Nt3* mRNA and proNT3 protein in the ipsilateral L4 DRG were increased by 2.0 (*P* < 0.01)- and 2.3 (*P* < 0.01)-fold, respectively, on day 7 post-SNL as compared to the corresponding sham mice (Fig. 1F–H).

Cellular distribution pattern of *Nt3* mRNA in injured DRG of CCI mice was examined, because commercially available proNT3/NT3 antibodies do not apply for immunohistochemistry staining and the expressional level of *Nt3* mRNA is extremely low in the DRG under normal conditions. ISHH assay followed by immunohistochemistry showed that *Nt3* mRNA was distributed predominantly in cellular cytoplasm and co-localized with β-tubulin III (a specific neuronal marker), but not with glutamine synthetase (GS, a marker for satellite glial cells), in the ipsilateral L3/4 DRG cells of sham or CCI mice (Fig. 1I), indicating that *Nt3* mRNA expresses exclusively in DRG neurons. Approximately 9.6 % of neurons in the ipsilateral L3/4 DRGs were positive for *Nt3* mRNA in sham mice on day 7 after surgery (Fig. 1J), of which about 1.98 % of DRG neurons were positive for small (< 500 μm^2^ in area), 3.14 % were medium (500 to 1,000 μm^2^ in area) and 4.48 % were large (>1,000 μm^2^ in area) (Fig. 1K). About 33.3 % of neurons in the ipsilateral L3/4 DRGs were positive for *Nt3* mRNA in CCI mice on day 7 after surgery (Fig. 1J), of which about 8.53 % of DRG neurons were positive for small, 10.02 % were medium and 14.75 % were large (Fig. 1K). Distribution pattern of *Nt3* mRNA and peripheral nerve trauma-induced increases of *Nt3* mRNA and proNT3 protein in injured DRGs strongly suggests the involvement of proNT3 in nerve trauma-induced neuropathic pain.

### 3.2. Preventing DRG proNT3 upregulation mitigates the development of nerve trauma-induced nociceptive hypersensitivities

**Fig. 2.**
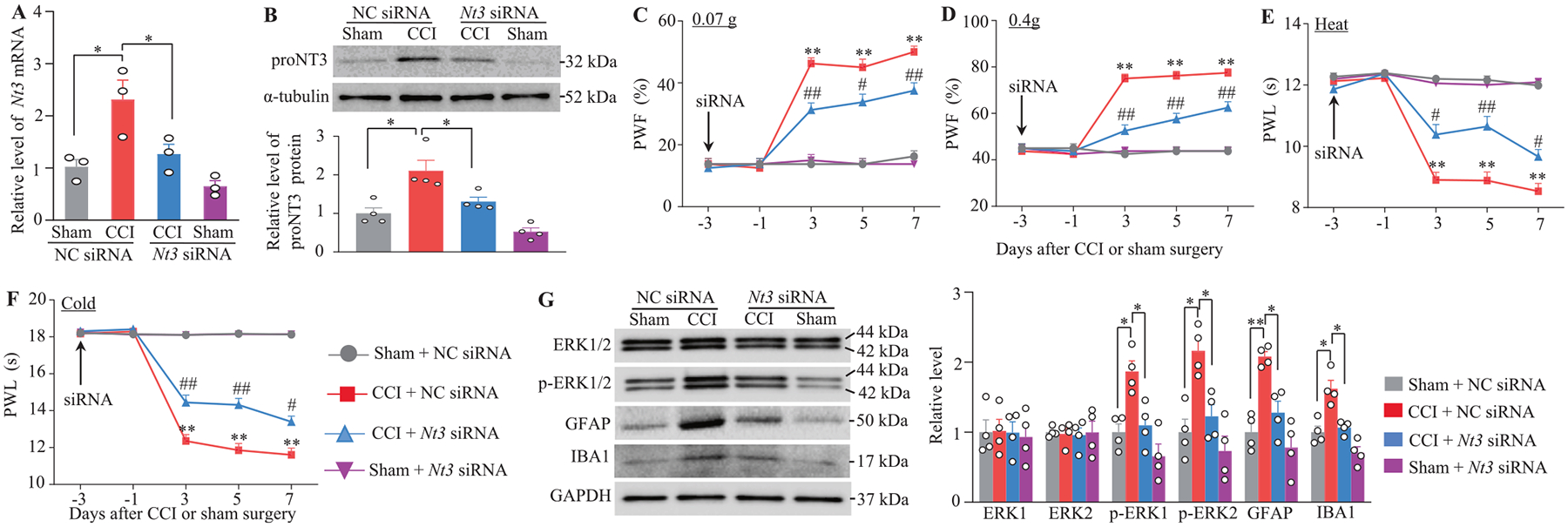
DRG microinjection of *Nt3* siRNA alleviates the development of nerve trauma-induced nociceptive hypersensitivity in male mice. (A and B) Expression of *Nt3* mRNA (A) and proNT3 protein (B) in the ipsilateral L3/4 DRGs on day 7 after CCI or sham surgery in mice with pre-microinjection of *Nt3* siRNA or negative control scrambled siRNA (NC siRNA) into unilateral L3/4 DRGs 3 days before CCI or sham surgery. n = 3–4 repeats (6–8 mice)/group. *P < 0.05 by two-way ANOVA followed by post hoc Tukey test. (C–F) Paw withdrawal frequencies (PWF) in response to 0.07 g (C) and 0.4 g (D) von Frey filament stimuli and paw withdrawal latencies (PWL) to heat (E) and cold (F) stimuli on the ipsilateral sides at the different days as indicated after CCI or sham surgery in mice with pre-microinjection of *Nt3* siRNA or NC siRNA into unilateral L3/4 DRGs 3 days before CCI or sham surgery. n = 8 mice/group. **P < 0.01 versus the NC siRNA plus sham group at the corresponding time points. #*P* < 0.05, ##*P* < 0.01 versus the NC siRNA plus CCI group at the corresponding time points by three-way ANOVA with repeated measures followed by post hoc Tukey test. (G) Expression of total ERK1/2, p-ERK1/2, GFAP and IBA1 proteins in the ipsilateral L3/4 spinal cord dorsal horn on day 7 after CCI or sham surgery in mice with pre-microinjection of *Nt3* siRNA or NC siRNA into unilateral L3/4 DRGs 3 days before CCI or sham surgery. n = 4 repeats (8 mice)/group. *P < 0.05, **P < 0.01 by two-way ANOVA followed by post hoc Tukey test.

To determine whether DRG proNT3 upregulation contributed to the development of nerve trauma-induced neuropathic pain, we prevented DRG proNT3 upregulation through single microinjection of *Nt3* short interfering RNA (*Nt3* siRNA; dissolved in PBS) into the ipsilateral L3/4 DRGs of adult male mice 3 days before sham or CCI surgery. Negative control scrambled siRNA (NC siRNA; dissolved in PBS) was used as a control. As expected, the levels of *Nt3* mRNA and proNT3 protein were increased by 2.3 (*P* < 0.01)- and 2.1 (*P* < 0.01)-fold, respectively, in the ipsilateral L3/4 DRGs 7 days after surgery in the CCI plus NC siRNA-treated group compared to those in the sham plus NC siRNA-treated group (Fig. 2A–B). These increases were significantly attenuated in the CCI plus *Nt3* siRNA-treated group (Fig. 2A–B). Basal levels of *Nt3* mRNA and proNT3 protein were not significantly changed in the ipsilateral L3/4 DRGs of the sham plus *Nt3* siRNA-treated group (Fig. 2A–B). Consistent with our previous studies (Wang et al., 2025; Wang et al., 2024; Zhang et al., 2024), CCI led to mechanical allodynia in response to mechanical stimuli (0.07 g and 0.4 g von Frey filaments) and heat and cold hyperalgesia in response to heat and cold stimuli, respectively, starting day 3 after surgery and persisting for at least 7 days on the ipsilateral (not contralateral) side in NC siRNA-microinjected male mice (Fig. 2C–F; Supplementary Fig. 2A–C). These nociceptive hypersensitivities were significantly alleviated in the CCI plus *Nt3* siRNA-treated group on days 3, 5 and 7 post-CCI (Fig. 2C–F). Microinjection of neither siRNA altered basal paw withdrawal responses on either side in sham male mice (Fig. 2C–F; Supplementary Fig. 2A–C) and locomotor function (Supplementary Table 2).

CCI-induced neuronal hyperexcitability in injured DRGs triggers the hyperactivation of neurons and glial cells in spinal cord dorsal horn through augmenting the release of neurotransmitters in primary afferents (Wen et al., 2023; Yang et al., 2021). To further confirm our behavioral observations above, we examined whether DRG microinjection of *Nt3* siRNA also affected the activations of spinal cord dorsal horn neurons, astrocytes and microglia, as documented by increases in the levels of phosphorylated extracellular signal-regulated kinase ½ (p-ERK1/2, a marker for neuronal hyperactivation), glial fibrillary acidic protein (GFAP, a marker for astrocyte hyperactivation) and ionized calcium-binding adaptor molecule 1 (IBA1, a marker for microglia hyperactivation), respectively, in male mice. The amounts of p-ERK1, p-ERK2, GFAP and IBA1 were increased by 1.9 (*P* < 0.05)-, 2.2 (*P* < 0.05)-, 2.1 (*P* < 0.01)- and 1.6 (*P* < 0.05)-fold, respectively, in the ipsilateral L3/4 dorsal horn on day 7 post-surgery in the CCI plus NC siRNA-treated group, as compared to those in sham plus NC siRNA-treated group (Fig. 2G). These increases were not observed in the CCI plus *Nt3* siRNA-treated mice (Fig. 2G). No significant differences in basal amounts of total ERK1/2 were seen in the ipsilateral L3/4 dorsal horn among all treated groups (Fig. 2G). Neither siRNA affected basal levels of p-ERK1/2, ERK1/2, GFAP and IBA1 in the ipsilateral L3/4 dorsal horn in sham mice (Fig. 2G).

Similar results were seen in the sham or CCI female mice with DRG microinjection of *Nt3* siRNA or NC siRNA (Supplementary Fig. 3A–I and Table 2).

**Fig. 3.**
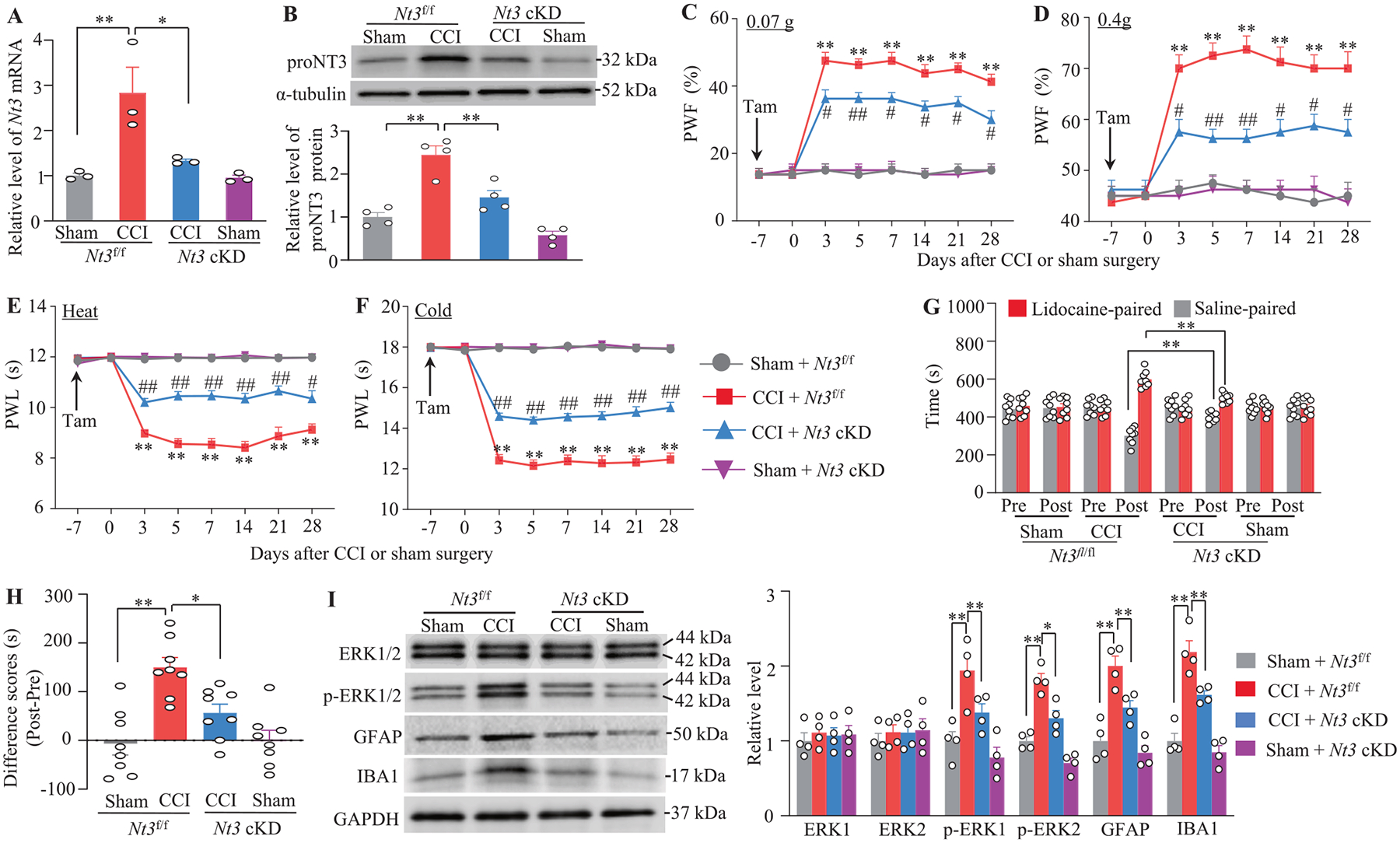
Genetic knockdown of *Nt3* gene in DRG sensory neurons attenuates the development of nerve trauma-induced nociceptive hypersensitivity in male mice. (A and B) Expression of *Nt3* mRNA (A) and proNT3 protein (B) in the ipsilateral L3/4 DRGs on day 28 after CCI or sham surgery in *Nt3*^f/f^ mice and *Nt3* cKD mice with pre-intraperitoneal injection of tamoxifen daily for 7 days. n = 3–4 repeats (6–8 mice)/group. *P < 0.05, **P < 0.01 by two-way ANOVA followed by post hoc Tukey test. (C–F) Paw withdrawal frequencies (PWF) in response to 0.07 g (C) and 0.4 g (D) von Frey filament stimuli and paw withdrawal latencies (PWL) to heat (E) and cold (F) stimuli on the ipsilateral sides at the different days as indicated after CCI or sham surgery in *Nt3*^f/f^ mice and *Nt3* cKD mice with pre-intraperitoneal injection of tamoxifen (Tam) daily for 7 days. n = 8 mice/group. **P < 0.01 versus the sham *Nt3*^f/f^ mice at the corresponding time points. #*P* < 0.05, ##*P* < 0.01 versus the CCI *Nt3*^f/f^ mice at the corresponding time points by three-way ANOVA with repeated measures followed by post hoc Tukey test. (G and H) Spontaneous nociceptive responses as assessed by the conditioned place preference (CPP) paradigm on day 21 after CCI or sham surgery in *Nt3*^f/f^ mice and *Nt3* cKD mice with pre-intraperitoneal injection of tamoxifen daily for 7 days. The time spent in each chamber (G) and difference scores for chamber preferences calculated by subtracting preconditioning (Pre) preference time from postconditioning (Post) time spent in the lidocaine-paired chamber (H). n = 8 mice/group. **P < 0.01 by two (H)- or three (G)-way ANOVA with repeated measures followed by post hoc Tukey test. (I) Expression of total ERK1/2, p-ERK1/2, GFAP and IBA1 proteins in the ipsilateral L3/4 spinal cord dorsal horn on day 28 after CCI or sham surgery in *Nt3*^f/f^ mice and *Nt3* cKD mice with pre-intraperitoneal injection of tamoxifen daily for 7 days. n = 4 repeats (8 mice)/group. *P < 0.05, **P < 0.01 by two-way ANOVA followed by post hoc Tukey test.

To exclude the possibilities that the behavioral phenotypes observed above may be caused by DRG microinjection-induced tissue damage and/or siRNA-produced off-targets, we prevented DRG proNT3 upregulation using male *Nt3* cKD mice, in which i.p. injection of tamoxifen (1 mg/mouse) daily for 7 consecutive days before CCI or sham surgery. Male *Nt3*^f/f^ mice were used as the control. As expected, the levels of *Nt3* mRNA and proNT3 protein were increased by 2.8 (*P* < 0.01)- and 2.4 (*P* < 0.01)-fold, respectively, in the ipsilateral L3/4 DRGs on day 28 post-surgery in tamoxifen-treated CCI *Nt3*^f/f^ mice, as compared to those in tamoxifen-treated sham *Nt3*^f/f^ mice (Fig. 3A–B). These increases were not observed in tamoxifen-treated CCI *Nt3* cKD mice (Fig. 3A–B). Basal levels of *Nt3* mRNA and proNT3 protein were not significantly altered in the ipsilateral L3/4 DRGs from tamoxifen-treated sham *Nt3* cKD mice (Fig. 3A–B). Tamoxifen-treated CCI *Nt3* cKD mice displayed impaired CCI-induced mechanical allodynia and heat and cold hyperalgesia on the ipsilateral side from day 3 to day 28 post-surgery (Fig. 3C–F). There were no changes in basal paw withdrawal responses on the contralateral side in tamoxifen-treated CCI *Nt3* cKD mice and on either side in tamoxifen-treated sham *Nt3* cKD mice or *Nt3*^f/f^ mice (Fig. 3C–F; Supplementary Fig. 3A–C). Unlike tamoxifen-treated CCI *Nt3*^f/f^ mice, *Nt3* cKD mice with tamoxifen injection failed to display significant preference toward either the saline- or lidocaine-paired chamber on day 28 post-CCI or sham surgery, indicating the alleviation in CCI-induced stimulation-independent spontaneous ongoing pain (Fig. 3G–H). As expected, the CCI-evoked increases of neuronal/astrocyte/microglia activations in the ipsilateral L3/4 dorsal horn from tamoxifen-injected *Nt3*^f/f^ mice were not observed in tamoxifen-injected *Nt3* cKD mice on day 28 following CCI (Fig. 3I). All siRNA-microinjected male and female mice and tamoxifen-treated *Nt3*^f/f^ male mice or *Nt3* cKD male mice exhibited normal locomotor activity (Supplementary Table 2).

Taken together, our findings indicate that DRG upregulated proNT3 may participate in the development of CCI-induced nociceptive hypersensitivity in both male and female mice.

### 3.3. Blocking DRG proNT3 upregulation alleviates the maintenance of nerve trauma-induced nociceptive hypersensitivities

**Fig. 4.**
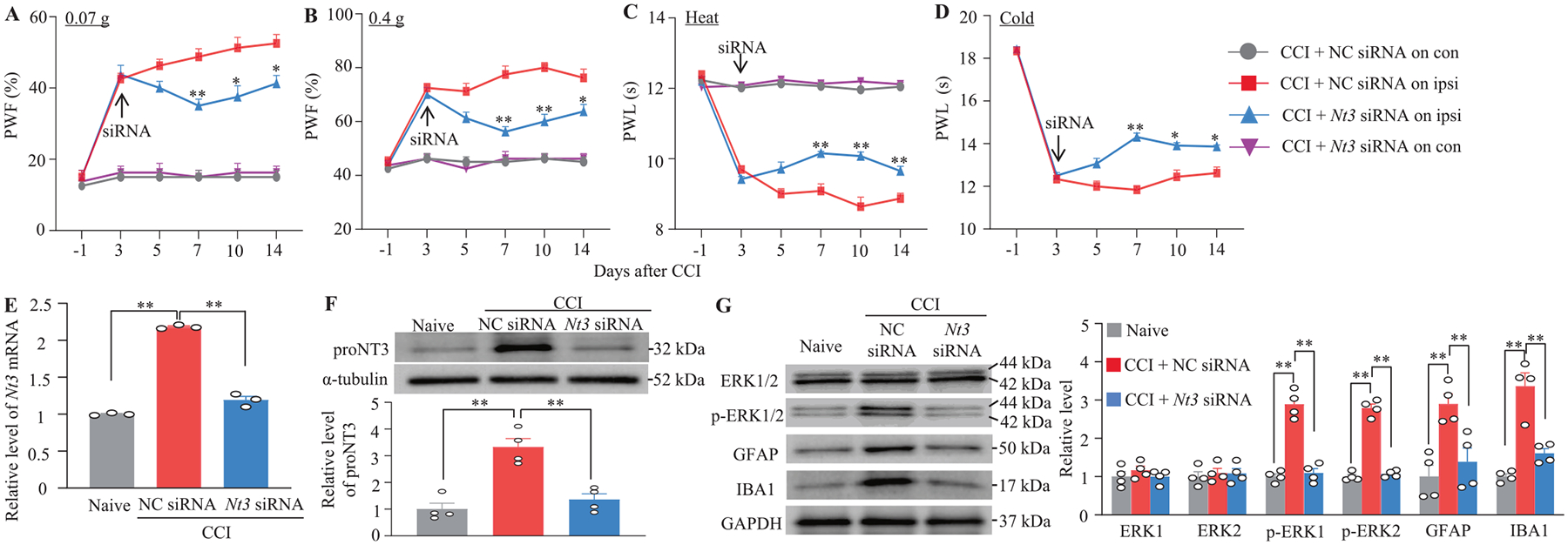
DRG microinjection of *Nt3* siRNA alleviates the maintenance of nerve trauma-induced nociceptive hypersensitivity in male mice. (A–D) Paw withdrawal frequencies (PWF) in response to 0.07 g (A) and 0.4 g (B) von Frey filament stimuli and paw withdrawal latencies (PWL) to heat (C) and cold (D) stimuli on the ipsilateral (ipsi) and contralateral (con) sides at the different days as indicated after CCI surgery in mice with post-microinjection of *Nt3* siRNA or negative control scrambled siRNA (NC siRNA) into the ipsilateral L3/4 DRGs 3 days after CCI surgery. n = 8 mice/group. *P < 0.05, **P < 0.01 versus the NC-siRNA plus CCI group at the corresponding time points by three-way ANOVA with repeated measures followed by post hoc Tukey test. (E and F) Expression of *Nt3* mRNA (E) and proNT3 protein (F) in the ipsilateral L3/4 DRGs on day 14 after CCI in mice with post-microinjection of *Nt3* siRNA or NC siRNA into the ipsilateral L3/4 DRGs 3 days after CCI surgery. n = 3–4 repeats (6–8 mice)/group. **P < 0.01 by two-way ANOVA followed by post hoc Tukey test. (G) Expression of total ERK1/2, p-ERK1/2, GFAP and IBA1 proteins in the ipsilateral L3/4 spinal cord dorsal horn on day 14 after CCI surgery in mice with post-microinjection of *Nt3*

To examine the role of DRG proNT3 upregulation in the maintenance of nerve trauma-induced neuropathic pain, we microinjected siRNA into the ipsilateral L3/4 DRGs 3 days after CCI in male mice, at this point when CCI-induced nociceptive hypersensitivities were significantly developed (Wang et al., 2025; Wang et al., 2024; Zhang et al., 2024). In the CCI plus NC-siRNA-treated group, mechanical allodynia and heat and cold hyperalgesia were observed on the ipsilateral side 5, 7, 10 and 14 days post-CCI (Fig. 4A–D). However, these nociceptive hypersensitivities were markedly attenuated on days 7, 10 and 14, but not on day 5, post-CCI in the CCI plus *Nt3*-siRNA-treated group (Fig. 4A–D). As expected, post-microinjection of neither siRNAs altered basal paw withdrawal responses on the contralateral side (Fig. 4A–C). Compared with naive mice, CCI increased the levels of *Nt3* mRNA and proNT3 protein by 2.2 (*P* < 0.01)- and 3.3 (*P* < 0.01)-fold, respectively, in the ipsilateral L3/4 DRGs on day 14 post-CCI in the CCI plus NC-siRNA-treated group (Fig. 4E–F), but these increases were not detected in the CCI plus *Nt3*-siRNA-treated group (Fig. 4E–F). Moreover, CCI-induced increases in the levels of p-ERK1, p-ERK2, GFAP and IBA1 (but not total ERK1 and 2) in the ipsilateral L3/4 dorsal horn on day 14 post-CCI from the CCI plus NC-siRNA-treated group were not observed in the CCI plus *Nt3*-siRNA-treated group (Fig. 4G). These findings suggest an important role of DRG upregulated proNT3 in the maintenance of CCI-induced pain hypersensitivities.

### 3.4. TrkC, but not TrkA and TrkB, mediates DRG proNT3 overexpression-induced nociceptive hypersensitivity

**Fig. 5.**
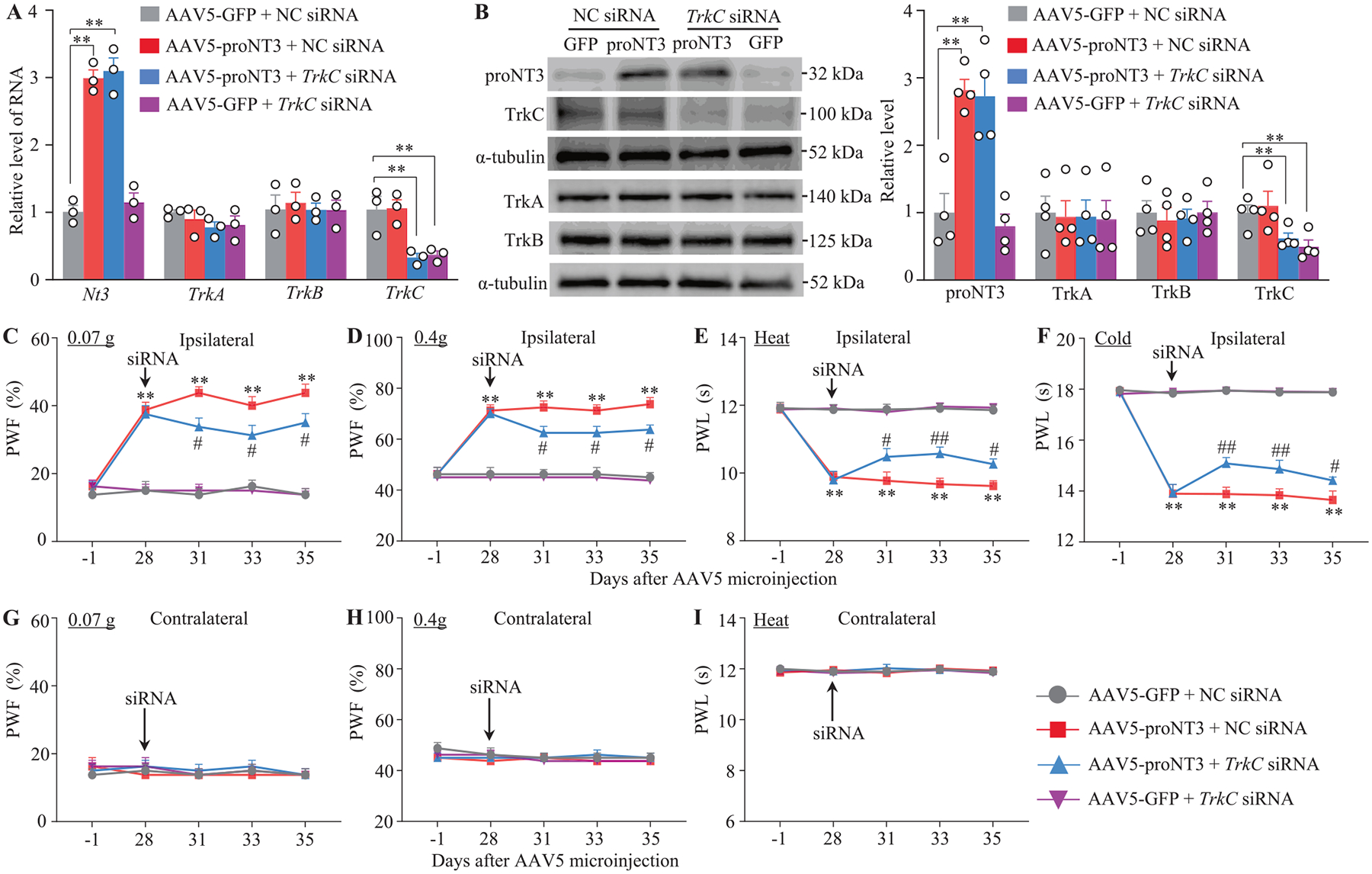
DRG TrkC knockdown mitigates DRG proNT3 overexpression-induced nociceptive hypersensitivity in male mice. (A and B) Expression of *Nt3*, *TrkA*, *TrkB* and *TrkC* mRNAs (A) and proNT3, TrkA, TrkB and TrkC proteins (B) in the ipsilateral L3/4 DRGs on day 35 after microinjection of AAV5-proNT3 or AAV5-GFP into the unilateral L3/4 DRGs in mice with post- microinjection of *TrkC* siRNA or negative control scrambled siRNA (NC siRNA) into the same DRGs 28 days after AAV5 microinjection. n = 3–4 repeats (6–8 mice)/group. **P < 0.01 by one-way ANOVA followed by post hoc Tukey test. (C–I) Paw withdrawal frequencies (PWF) in response to 0.07 g (C and G) and 0.4 g (D and H) von Frey filament stimuli and paw withdrawal latencies (PWL) to heat (E and I) and cold (F) stimuli on the ipsilateral (C–F) and contralateral (G–I) sides at the different days as indicated after microinjection of AAV5-proNT3 or AAV5-GFP into the unilateral L3/4 DRGs in mice with post-microinjection of *TrkC* siRNA or NC siRNA into the same DRGs 28 days after AAV5 microinjection. n = 8 mice/group. **P < 0.01 versus the AAV5-GFP plus NC siRNA-treated group at the corresponding days. #*P* < 0.05, ##*P* < 0.01 versus the AAV5-proNT3 plus NC-siRNA-treated group at the corresponding time points. Two-way ANOVA with repeated measures followed by post hoc Tukey test.

**Fig. 6.**
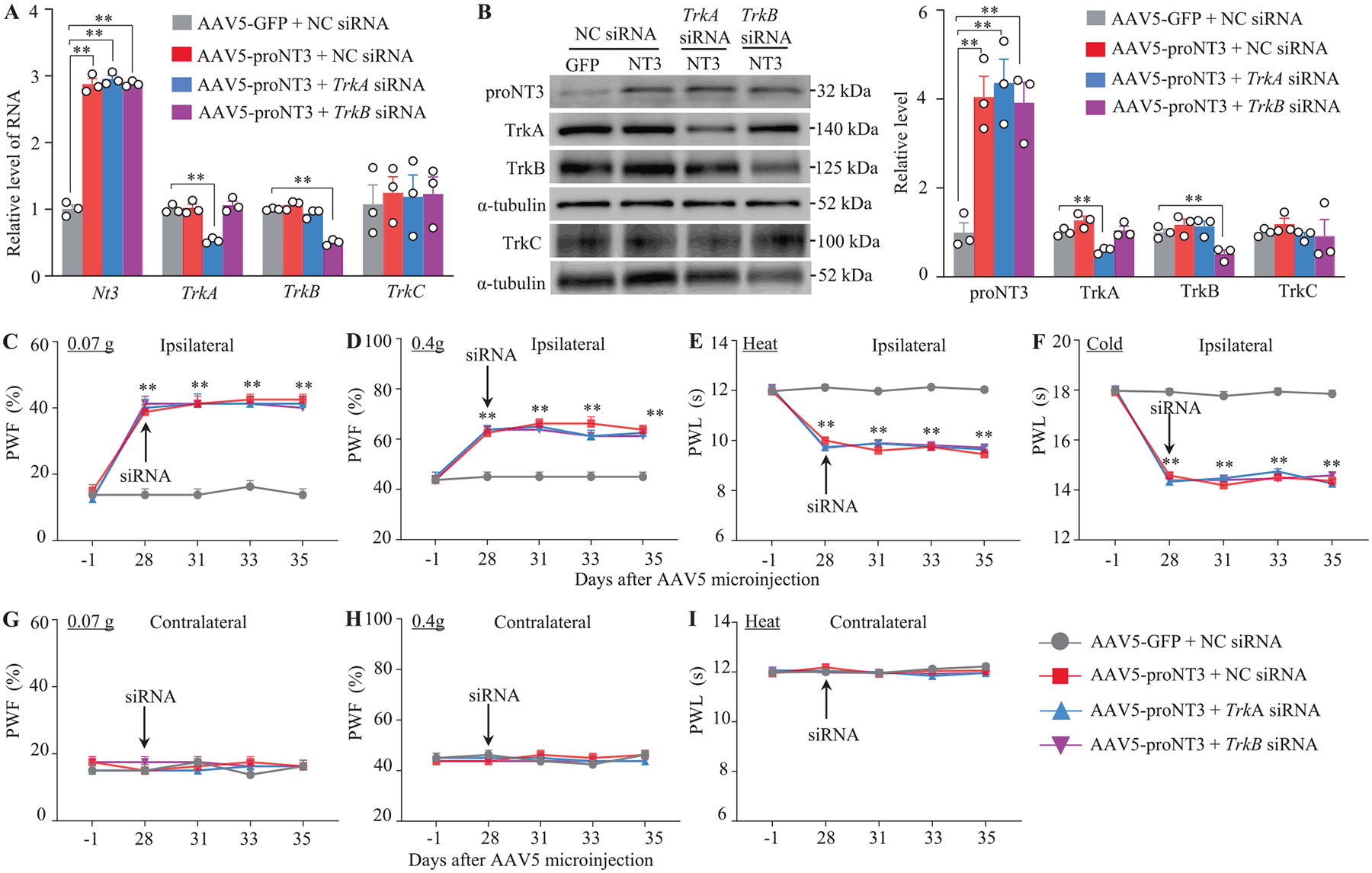
DRG TrkA or TrkB knockdown does not affect DRG proNT3 overexpression-induced nociceptive hypersensitivity in male mice. (A and B) Expression of *Nt3*, *TrkA*, *TrkB* and *TrkC* mRNAs (A) and proNT3, TrkA, TrkB and TrkC proteins (B) in the ipsilateral L3/4 DRGs on day 35 after microinjection of AAV5-proNT3 or AAV5-GFP into the unilateral L3/4 DRGs in mice with post-microinjection of *TrkA* siRNA, *TrkB* siRNA, or negative control scrambled siRNA (NC siRNA) into the same DRGs 28 days after AAV5 microinjection. n = 3 repeats (6 mice)/group. **P < 0.01 by one-way ANOVA followed by post hoc Tukey test. (C–I) Paw withdrawal frequencies (PWF) in response to 0.07 g (C and G) and 0.4 g (D and H) von Frey filament stimuli and paw withdrawal latencies (PWL) to heat (E and I) and cold (F) stimuli on the ipsilateral (C–F) and contralateral (G–I) sides at the different days as indicated after microinjection of AAV5-proNT3 or AAV5-GFP into the unilateral L3/4 DRGs in mice with post-microinjection of *TrkA* siRNA, *TrkB* siRNA or NC siRNA into the same DRGs 28 days after AAV5 microinjection. n = 8 mice/group. **P < 0.01 versus the AAV5-GFP plus NC siRNA-treated group at the corresponding days by two-way ANOVA with repeated measures followed by post hoc Tukey test.

Microinjection of AAV5 expressing full-length *Nt3* mRNA (AAV5-proNT3) into unilateral L3/4 DRGs in the NC-siRNA-treated male mice produced substantial increases in the amounts of *Nt3* mRNA and proNT3 protein in microinjected L3/4 DRGs on day 35 post-viral microinjection (Fig. 5A–B and Fig. 6A–B) and neuropathic pain-like symptoms (Fig. 5C–F and Fig. 6C–F). Mechanical allodynia and heat and cold hyperalgesia were fully developed 28 days post-AAV5-proNT3 microinjection and persisted for at least 35 days on the ipsilateral (not contralateral) side (Fig. 5C–I and Fig. 6C–I). To examine whether these nociceptive hypersensitivities were mediated by TrkA, TrkB or TrkC in DRG, we microinjected their respective siRNAs into the ipsilateral L3/4 DRGs on day 28 after AAV5 microinjection. These siRNAs specifically and significantly knocked down the expression of mRNA and protein of the corresponding TrkC (Fig. 5A–B), TrkA and TrkB (Fig. 6A–B), respectively, in the microinjected DRGs. Behaviorally, the increases in paw withdrawal frequencies to 0.07 g and 0.4 g von Frey filament stimuli and decreases in paw withdrawal latencies to heat and cold stimuli on the ipsilateral side in the AAV5-proNT3 plus NC siRNA-treated group were impaired in the AAV5-proNT3 plus *TrkC* siRNA-treated group on days 31, 33 and 35 post-viral microinjections (Fig. 5C–F). Interestingly, DRG microinjection of neither *TrkA* siRNA nor *TrkB* siRNA affected the AAV5-proNT3-induced mechanical allodynia and heat and cold hyperalgesia during the observation period (Fig. 6C–F). None of these siRNAs altered basal responses to mechanical, heat and cold stimuli on the contralateral side of the AAV5-proNT3-treated group and on both sides of the AAV5-GFP-treated group (Fig. 5C–I and Fig. 6C–I). Locomotor functions were normal among treatment groups (Supplementary Table 2). These data suggest that proNT3 produces nociceptive hypersensitivity primarily through the activation of TrkC, but not TrKA and TrkB, in the DRG neurons.

### 3.5. Intrathecal NT3 protein impairs the CCI- or DRG proNT3 overexpression-induced mechanical allodynia and heat hyperalgesia

**Fig. 7.**
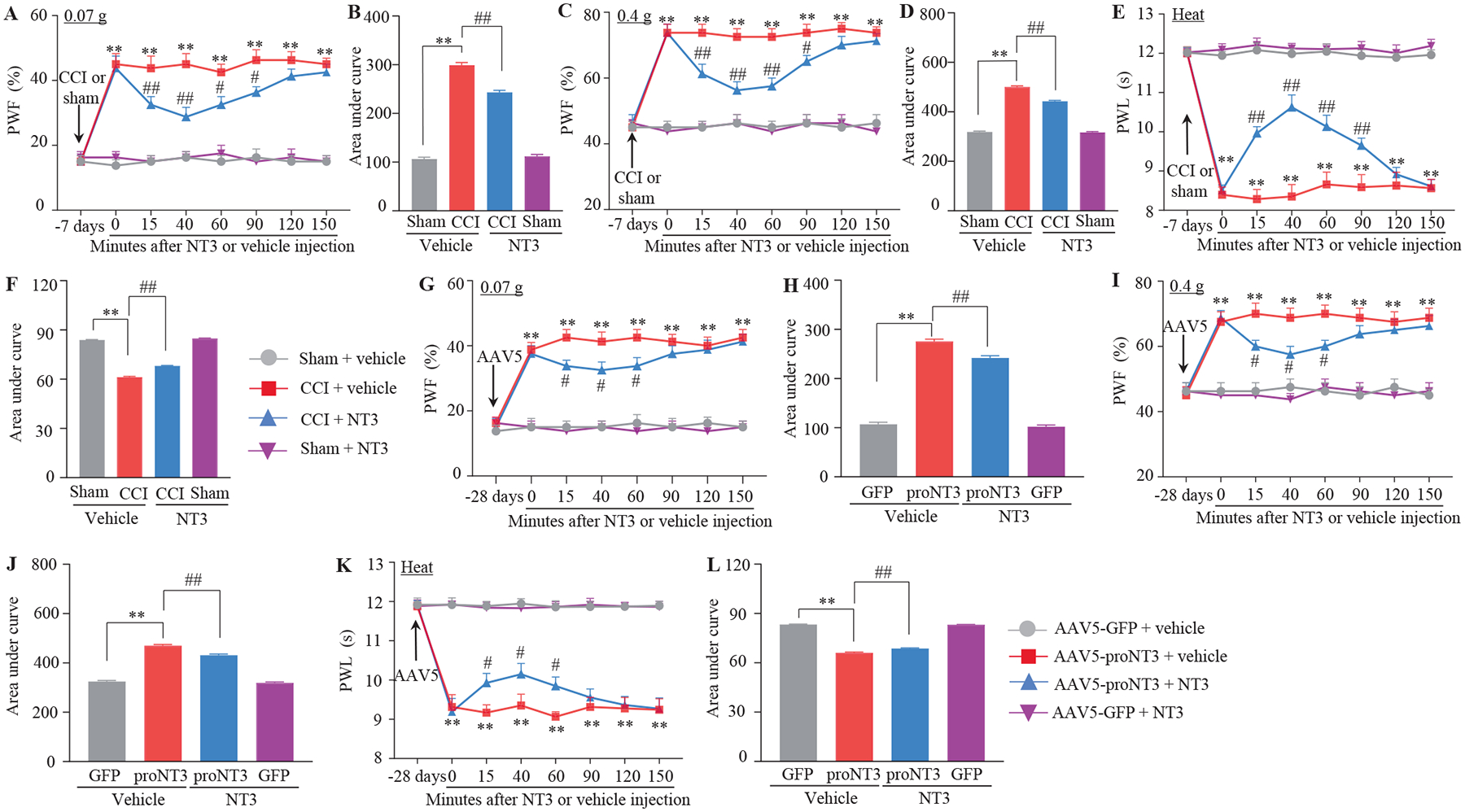
Intrathecal injection of NT3 alleviates mechanical allodynia and heat hyperalgesia caused by peripheral nerve injury or DRG proNT3 overexpression in male mice. (A–F) Paw withdrawal frequencies (PWF) in response to 0.07 g (A) and 0.4 g (C) von Frey filament stimuli and paw withdrawal latencies (PWL) to heat stimulation (E) on the ipsilateral sides at the different time points as indicated after intrathecal injection of NT3 or vehicle in mice with pre-CCI or sham surgery 7 days before intrathecal injection. B, D and F: the quantifications of the corresponding areas under the curves for A, C and E, respectively. n = 8 mice/group. **P <0.01 versus sham plus vehicle group at the corresponding time points. #*P* < 0.05, ##*P* < 0.01 versus CCI plus vehicle group at the corresponding time points. Two (B, D and F)- or three (A, C and E)-way ANOVA with repeated measures followed by post hoc Tukey test. (G–L) Paw withdrawal frequencies (PWF) in response to 0.07 g (G) and 0.4 g (I) von Frey filament stimuli and paw withdrawal latencies (PWL) to heat stimulation (K) on the ipsilateral sides at the different time points as indicated after intrathecal injection of NT3 or vehicle in mice with pre-microinjection of AAV5-proNT3 (proNT3) or AAV5-GFP (GFP) into the unilateral L3/4 DRGs 28 days before intrathecal injection. H, J and L: the quantifications of the corresponding areas under the curves for G, I and K, respectively. n = 8 mice/group. **P < 0.01 versus AAV5-GFP plus vehicle group at the corresponding time points. #*P* < 0.05, ##*P* < 0.01 versus AAV5-proNT3 plus vehicle group at the corresponding time points. One (H, J and L)- or two (G, I and K)-way with repeated measures followed by post hoc Tukey test.

Considering that proneurotrophins and mature neurotrophins exert opposing role in central nervous system (Gibon and Barker, 2017), we examined whether i.th. injection of NT3 protein affected the established nociceptive hypersensitivity caused by CCI- or DRG proNT3 overexpression. I.th. administration of NT3 at 2 μg on day 7 post-CCI significantly blocked the CCI-induced increases in paw withdrawal frequency to 0.07 and 0.4 g von Frey filament stimuli and the CCI-induced reduction in paw withdrawal latency to heat stimulation starting 15 min after i.th. injection and lasting for 90 min on the ipsilateral side (Fig. 7A–F). Additionally, i.th. injection of NT3 at the same dosage on day 28 post-AAV5 microinjection markedly mitigated the AAV5-proNT3-induced increases in paw withdrawal frequency to 0.07 and 0.4 g von Frey filament stimuli and the AAV5-proNT3-induced reduction in paw withdrawal latency to heat stimulation starting 15 min after i.th. injection and lasting for 60 min on the ipsilateral side (Fig. 7G–L). NT3 at 2 μg did not affect basal behavioral responses of sham mice or the AAV5-GFP-microinjected mice on the ipsilateral side (Fig. 7A–L) and locomotor function (Supplementary Table 2). These findings highlight the potential of NT3 as an effective therapeutic agent for neuropathic pain.

### 3.6. Upregulated proNT3 contributes to the CCI-induced increase of CCL2 through TrkC-mediated signaling in injured DRG

**Fig. 8.**
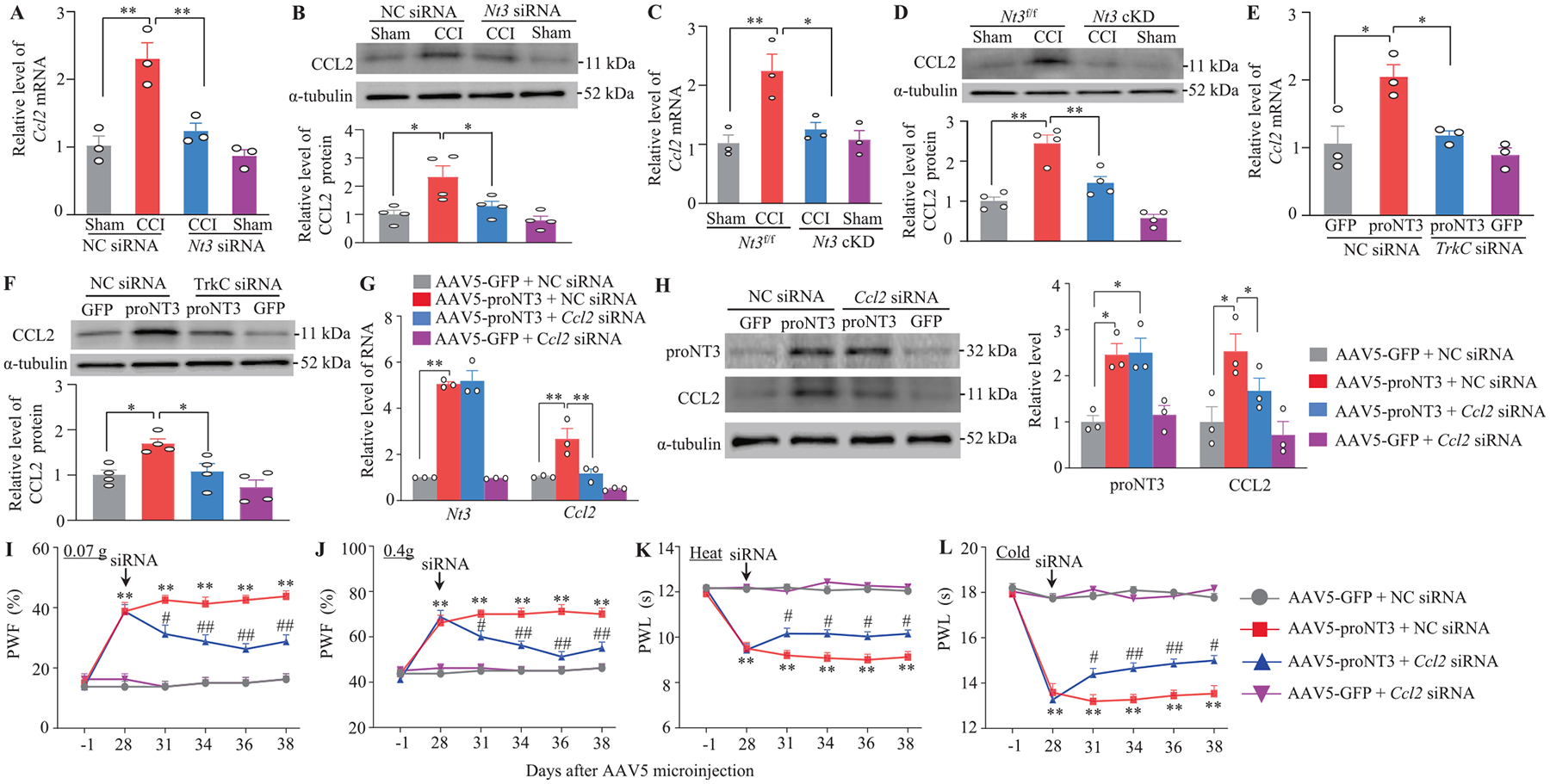
Upregulated proNT3 in the injured DRG contributes to nerve trauma-induced CCL2 expression via TrkC-mediated signaling. (A and B) Expression of *Ccl2* mRNA (A) and CCL2 protein (B) in the ipsilateral L3/4 DRGs on day 7 after CCI or sham surgery in male mice with pre-microinjection of *Nt3* siRNA or negative control scrambled siRNA (NC siRNA) into unilateral L3/4 DRGs 3 days before CCI or sham surgery. n = 3–4 repeats (6–8 mice)/group. *P < 0.05, **P < 0.01 by two-way ANOVA followed by post hoc Tukey test. (C and D) Expression of *Ccl2* mRNA (C) and CCL2 protein (D) in the ipsilateral L3/4 DRGs on day 28 after CCI or sham surgery in male *Nt3*^f/f^ mice and *Nt3* cKD mice with pre-intraperitoneal injection of tamoxifen daily for 7 days. n = 3–4 repeats (6–8 mice)/group. *P < 0.05, **P < 0.01 by two-way ANOVA followed by post hoc Tukey test. (E and F) Expression of *Ccl2* mRNA (E) and CCL2 protein (F) in the ipsilateral L3/4 DRGs on day 35 after microinjection of AAV5-proNT3 or AAV5-GFP into the unilateral L3/4 DRGs in male mice with post-microinjection of *TrkC* siRNA or NC siRNA into the same DRGs 28 days after AAV5 microinjection. n = 3 repeats (6 mice)/group. *P < 0.05 by one-way ANOVA followed by post hoc Tukey test. (G and H) Expression of *Nt3* and *Ccl2* mRNAs (G) as well as proNT3 and CCL2 proteins (H) in the ipsilateral L3/4 DRGs on day 38 after microinjection of AAV5-proNT3 (proNT3) or AAV5-GFP (GFP) into the unilateral L3/4 DRGs in male mice with post-microinjection of *Ccl2* siRNA or NC siRNA into the same DRGs 28 days after AAV5 microinjection. n = 3 repeats (6 mice)/group. *P < 0.05, **P < 0.01 by one-way ANOVA followed by post hoc Tukey test. (I–L) Paw withdrawal frequencies (PWF) in response to 0.07 g (I) and 0.4 g (J) von Frey filament stimuli and paw withdrawal latencies (PWL) to heat (K) and cold (L) stimuli on the ipsilateral sides at the different days as indicated after microinjection of AAV5-proNT3 (proNT3) or AAV5-GFP (GFP) into the unilateral L3/4 DRGs in male mice with post-microinjection of *Ccl2* siRNA or NC siRNA into the same DRGs 28 days after AAV5 microinjection. n = 8 mice/group. **P < 0.01 versus the AAV5-GFP plus NC-siRNA-treated group at the corresponding time points. #*P* < 0.05, ##*P* < 0.01 versus the AAV5-proNT3 plus NC-siRNA-treated group at the corresponding time points by two-way ANOVA with repeated measures followed by post hoc Tukey test.

Lastly, we explored how DRG upregulated proNT3 contributed to the CCI-induced neuropathic pain. CCL2 is a key player in the genesis of nerve trauma-induced neuropathic pain (Biber and Boddeke, 2014; Zhang et al., 2017). Given that CCL2 might act as a downstream signal of the proNT3/TrkC pathway (Salvador et al., 2014), we reasoned that CCL2 likely mediated the role of DRG upregulated pro-NT3 in CCI-induced neuropathic pain. Consistent with previous studies (Grace et al., 2016; Wang et al., 2023; Wen et al., 2023), the amounts of *Ccl2* mRNA and CCL2 protein were significantly elevated in the ipsilateral L3/4 DRGs on days 7 and 14 post-CCI from the NC-siRNA-treated CCI male (Fig. 8A–B and Supplementary Fig. 5A–B) and female (Supplementary Fig. 6) mice or on day 28 post-CCI from the tamoxifen-treated CCI *Nt3*^f/f^ male mice (Fig. 8C–D). These elevations were substantially attenuated in the CCI plus *Nt3* siRNA-treated male (Fig. 8A–B and Supplementary Fig. 5A–B) and female (Supplementary Fig. 6) mice or in the CCI plus tamoxifen-treated *Nt3* cKD male mice (Fig. 8C–D). More importantly, microinjection of AAV5-proNT3, but not AAV5-GFP, into the unilateral L3/4 DRGs significantly increased the levels of *Ccl2* mRNA and CCL2 protein in the ipsilateral L3/4 DRGs 35 days after AAV5 microinjection in the NC siRNA-treated mice (Fig. 8E–H). These increases were blocked in the AAV5-proNT3 plus *TrkC* siRNA- or *Ccl2* siRNA-treated mice, although DRG microinjection of these siRNAs did not alter basal levels of *Ccl2* mRNA and CCL2 protein in the AAV5-GFP-microinjected DRG (Fig. 8E–H). Moreover, DRG microinjection of *Ccl2* siRNA noticeably alleviated the AAV5-proNT3-induced increases in paw withdrawal frequencies to 0.07 g and 0.4 g von Frey filament stimuli and the AAV5-proNT3-induced decreases in paw withdrawal latencies to heat and cold stimuli on the ipsilateral side on days 31, 34, 36 and 38 post-viral microinjections (Fig. 8I–L). In addition, the AAV5-proNT3-induced increases of neuronal/astrocyte/microglia activations in the ipsilateral L3/4 dorsal horn from the AAV5-proNT3 plus NC-siRNA-treated mice were not observed in the AAV5-proNT3 plus *Ccl2*-siRNA-treated mice on day 38 post-viral microinjection (Supplementary Fig. 7). As expected, DRG microinjection of *Ccl2* siRNA did not change basal behavioral responses on the contralateral side (Supplementary Fig. 8A–C) and locomotor activity (Supplementary Table 2). Given that *Nt3* mRNA co-expressed with *TrkC* and *Ccl2* mRNAs in individual small, medium and large DRG neurons (Supplementary Fig. 9), our data suggest that upregulated proNT3 participates in CCI-induced nociceptive hypersensitivity at least in part due to the TrkC-mediated activation of *Ccl2* gene expression in DRG neurons.

## 4. Discussion

Previous studies have attributed the induction and maintenance of nerve trauma-induced neuropathic pain at least in part to nerve trauma-induced increase of CCL2 in the injured DRG (Biber and Boddeke, 2014; Zhang et al., 2017). However, how this increase occurs after peripheral nerve trauma is still incompletely understood. Here we reported that the upregulation of proNT3 temporally correlated with the increase of CCL2 in the injured DRG after peripheral nerve trauma. The upregulated proNT3 contributes to the development and maintenance of nerve trauma-induced neuropathic pain through promoting TrkC-mediated activation of *Ccl2* gene in neurons of the injured DRG. It is likely that proNT3 plays a pivotal role in nerve trauma-induced nociceptive hypersensitivity.

proNT3 and its mature form NT3 have distinct expression patterns in the DRG. Recent (Sharma et al., 2025) and current studies using an antibody that recognizes both NT3 and proNT3 demonstrated that only proNT3, but not NT3, was detected in the DRG under both normal and nerve trauma/chemotherapy-induced neuropathic pain conditions. Given that NT3 is the C-terminal portion of proNT3 (Teng et al., 2010; Yano et al., 2009), the increases in NT3-like immunoreactivities in the DRG following resiniferatoxin treatment or removal of adjacent DRG reported in previous studies (Tender et al., 2011; Wang et al., 2008) are, in fact, proNT3 rather than NT3. Upregulated *Nt3* mRNA in injured DRG neurons following peripheral nerve trauma (Kazemi et al., 2017; Wang et al., 2008) is actually translated into proNT3 protein. The lack of proNT3 cleavage into mature NT3 in the DRG is intriguing. Whether this is due to the absence or low expression of furin and other proconvertases in the DRG remains to be determined in our future study.

Our quantitative RT-PCR and Western blot assays showed significant and time-dependent increases of *Nt3* mRNA and proNT3 in the injured DRG. These increases are correlated with the development and maintenance of nerve trauma-induced nociceptive hypersensitivity. Due to the limitations of commercially available NT3 antibodies for immunohistochemistry, we employed the ISHH assay to examine the cellular localization of *Nt3* mRNA. Our findings indicate that nerve trauma-induced *Nt3* mRNA upregulation occurred exclusively in neurons of the injured DRG. These findings suggest that DRG upregulated proNT3 plays a critical role in nerve trauma-induced nociceptive hypersensitivity. It remains unclear how *Nt3* gene is transcriptionally activated under neuropathic pain conditions. Potential contributions of transcription factor activation, epigenetic modifications, or enhanced *Nt3* RNA stability to the nerve trauma-induced upregulation of *Nt3* mRNA in the DRG remain to be clarified in future studies.

The upregulated proNT3 is required for nerve trauma-induced nociceptive hypersensitivity. Previous studies have shown that intrathecal injection of *Nt3* ASO or local/systemic administration of NT3 antiserum alleviated nerve trauma-induced mechanical allodynia (Deng et al., 2000; Quintao et al., 2008; White, 2000; Zhou et al., 2000b). Given that DRG expresses only proNT3, not mature NT3, these studies indicate that the contribution of DRG proNT3 to nerve trauma-induced mechanical allodynia is likely attributed to *Nt3* ASO/NT3 antiserum-caused blockage of proNT3 in the injured DRG. The present study further demonstrated that blocking CCI-induced increase of DRG proNT3 through either the microinjection of *Nt3* siRNA into the ipsilateral L3/4 DRGs or i.p. injection of tamoxifen in the *Nt3* cKD mice attenuated the development and maintenance of CCI-induced nociceptive hypersensitivity. Unexpectedly, neither of these genetic knockdown strategies affected basal proNT3 expression. While the underlying cause remains unclear, it is plausible that the relatively low baseline expression of *Nt3* mRNA under normal conditions limits the efficacy of *Nt3* siRNA or tamoxifen at the current dosing regimen. Importantly, mimicking nerve trauma-induced upregulation of DRG proNT3 through DRG microinjection of AAV5-proNT3 led to neuropathic pain-like symptoms in naïve mice. These symptoms are mediated by TrkC, but not by TrkA, TrkB and p75 NTR (Sharma et al., 2025). Consistent with previous studies (Sharma et al., 2025; Yano et al., 2009), proNT3, like NT3, exerts its biological effects through TrkC activation, because mature NT3 is generated from the C-terminal portion of proNT3. Intrathecal injection of NT3 protein mitigated mechanical allodynia and heat hyperalgesia induced by CCI or DRG proNT3 overexpression, as observed in both the present study and previous work (Wilson-Gerwing et al., 2005). The antinociceptive effect of exogenous NT3 is likely mediated through competitive binding with endogenous proNT3 at TrkC. Interestingly, earlier studies reported that intrathecal NT3 induced mechanical allodynia in rats (White et al., 1998), and that direct delivery of NT3 into the DRG did not affect baseline responses to mechanical stimulation (Zhou et al., 2000a). These opposing or inconsistent findings may be attributed to the differences in NT3 dosage and/or the animal species used. Taking together, our findings indicate that DRG upregulated proNT3 is both necessary and sufficient for nerve trauma-induced neuropathic pain through triggering TrkC activation in the injured DRG.

CCL2 functions as a downstream effector of proNT3-triggered TrkC activation in the injured DRG under the conditions of nerve trauma-induced neuropathic pain. CCI-induced increases in the levels of *Ccl2* mRNA and CCL2 protein in the ipsilateral L3/4 DRGs were prevented by DRG microinjection of *Nt3* siRNA or tamoxifen injection in *Nt3* cKD mice. Conversely, DRG overexpression of proNT3 elevated the basal levels of *Ccl2* mRNA and CCL2 protein in the DRG. These elevations could be blocked by DRG co-microinjection of *TrkC* siRNA or *Ccl2* siRNA. DRG microinjection of *Ccl2* siRNA also alleviated nociceptive hypersensitivity caused by DRG overexpression of proNT3. Given that *Nt3* mRNA co-expressed with *TrkC* and *Ccl2* mRNAs in DRG neurons, upregulated proNT3 promotes the nerve trauma–induced increases in *Ccl2* mRNA and CCL2 protein, likely mediated by TrkC activation in injured DRG neurons. How proNT3/TrkC activation leads to transcriptional activation of the *Ccl2* gene promoter in injured DRG neurons remains unclear. TrkC activation activates PI3K-Akt signaling, resulting in transcriptional activity of the nuclear factor-κB (NFκB) (Bai et al., 2009; Reichardt, 2006). TrkC activation also activates Ras–Raf–MEK–ERK signaling to phosphorylates c-Fos and c-Jun, promoting AP-1 complex formation (Nagesh et al., 2021; Reichardt, 2006). Considering that *Ccl2* promoter contains the binding motifs of both AP-1 and NFκB (Deng et al., 2013), we will examine whether these intracellular signals mediate the role of proNT3/TrkC in nerve injury-induced CCL2 increase in injured DRG neurons in our future study.

Despite extensive investigation, the molecular mechanisms underlying nerve trauma–induced neuropathic pain remain incompletely understood. Small, medium and large DRG neurons exhibit unique characteristics and function under neuropathic pain conditions. Early studies showed that knockdown/knockout of Nav1.7, 1.8 or 1.9 mainly expressed in small DRG neurons or selectively ablating most nociceptors (80 %) in mouse DRG did not affect the development and maintenance of neuropathic pain (Akopian et al., 1999; Amaya et al., 2006; Kerr et al., 2001; Nassar et al., 2005; Priest et al., 2005). Recent works demonstrated that peripheral nerve trauma increased excitability and decreased rheobase in injured small DRG neurons (Gong et al., 2014). Blocking upregulation of GPR151 or *SS-lncRNA* mainly expressed in small DRG neurons attenuated nerve trauma-induced neuropathic pain and DRG neuronal hyperexcitability (Wang et al., 2023; Xia et al., 2021). The evidence suggests involvement of small DRG neurons in neuropathic pain. It is well proved that medium/large DRG neurons undergo functional changes following nerve trauma and contribute to neuropathic pain. The increased spontaneous ectopic activity was found in injured myelinated afferents (but not un-myelinated C-fibers) and corresponding medium/large DRG neurons (Liu et al., 2000a; Liu et al., 2000b; Tal et al., 1999). Mechanical allodynia after nerve trauma was mediated by Aβ fibers (Duan et al., 2014; Xu et al., 2015). After oxaliplatin injection, bursts of action potentials induced by the cooling were identified in mouse and human myelinated A fibers, but not unmyelinated C-fibers (Sittl et al., 2012). Silencing pain-associated genes (e.g., Nav 1.6, *Kcan2* antisense RNA, *NIS-lncRNA*) mainly expressed in medium/large DRG neurons mitigated neuropathic pain and prevented abnormal spontaneous activity in injured medium/large DRG neurons (Du et al., 2022; Xie et al., 2015; Zhao et al., 2013). CGRP and SP in injured myelinated fibers and medium/large DRG neurons were markedly increased as early as 2 days after nerve trauma (Devor, 2009; Weissner et al., 2006). Our ISHH assay showed *Nt3* mRNA upregulation occurred in all three types of DRG neurons of injured DRG. The increased proNT3 promotes CCL2 production through TrkC likely via autocrine/paracrine mechanisms in injured DRG neurons. Given that CCL2 directly excites small, medium and large DRG neurons (Jung et al., 2008; Sun et al., 2006; Van et al., 2011; White et al., 2005), our findings strongly suggest that DRG increased proNT3 contributes to neuropathic pain genesis by promoting TrkC-mediated, CCL2-triggered hyperexcitability in small, medium and large neurons of injured DRG.

In summary, our study reveals a novel mechanism whereby upregulated proNT3 promotes TrkC-dependent CCL2 expression in the injured DRG neurons under conditions of nerve trauma-induced neuropathic pain. Given that blockage of upregulated proNT3 in the injured DRG attenuated nerve trauma-induced neuropathic pain without impacting basal or acute nociception or locomotor activity, proNT3 may represent a promising therapeutic target for this disorder. However, given that proNT3 is expressed in multiple tissues beyond the DRG, potential side effects associated with targeting proNT3 should be carefully considered.

## Supplementary Material

1

## Data Availability

Data will be made available on request.

## Appendix A. Supplementary data

Supplementary data to this article can be found online at https://doi.org/10.1016/j.bbi.2026.106256.
