# Supplementary material for: Contribution of proneurotrophin-3 to nerve trauma-induced neuropathic pain through promoting TrkC-mediated increase of CCL2 in primary sensory neurons
Source: Brain Behav Immun. Author manuscript; Available in PMC 2026 Jun 26. (PMC13307115; doi:10.1016/j.bbi.2026.106256)
Supplement: 1 [file NIHMS2189222-supplement-1.pdf]

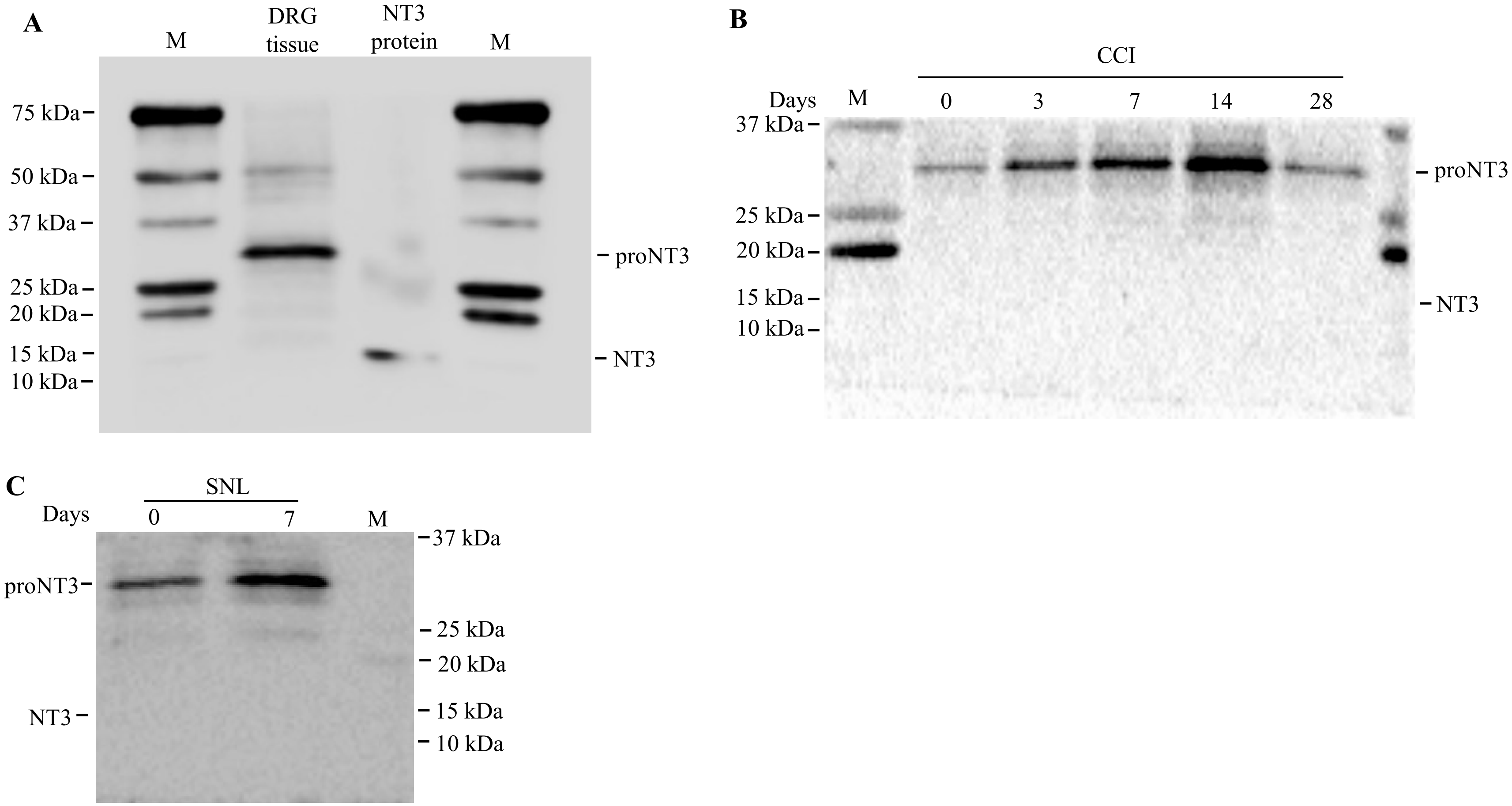

Supplementary Figure 1. proNT3, but not mature NT3, is detected in naïve DRG and injured DRG. (A) Western blot analysis of total cellular lysates from naïve mouse DRG and recombinant NT3 standard (SinoBiological) using an anti-NT3 antibody (ANT-003, Alomone Labs) demonstrates that the antibody detects both proNT3 (~32 kDa) and mature NT3 (~14.5 kDa). Only proNT3 is detected in naïve DRG lysates. (B) proNT3 expression is time-dependently upregulated in the ipsilateral L3/4 DRG following CCI. Mature NT3 is not detected in the ipsilateral L3/4 DRG at 0, 3, 7, 14, or 28 days post-CCI. (C) proNT3 expression is upregulated in the ipsilateral L4 DRG 7 days after SNL compared to naïve mice (0 day). Mature NT3 is not detected in the ipsilateral L4 DRG on days 0 and 7 after SNL. M: Molecular weight marker.

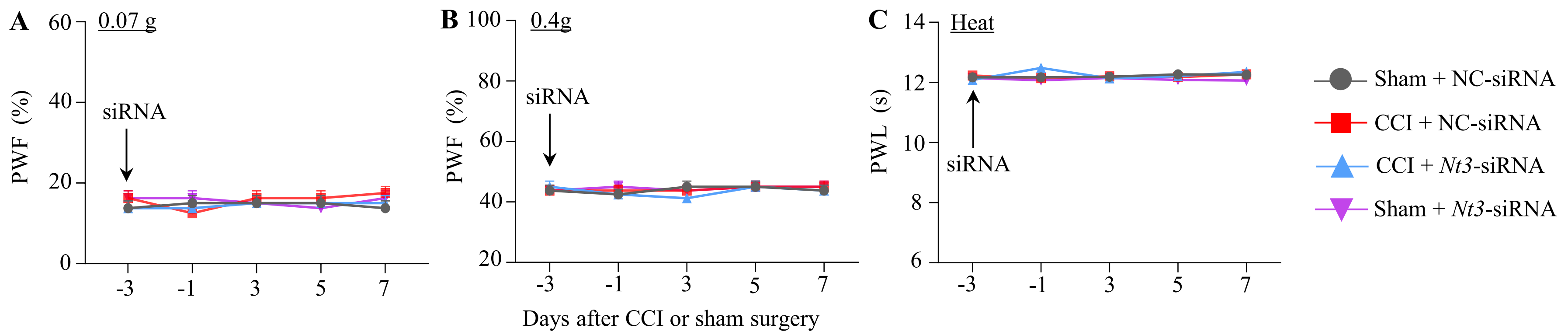

Supplementary Figure 2. DRG microinjection of *Nt3* siRNA does not alter basal behavioral response on the contralateral side in CCI mice. (A-C) Paw withdrawal frequencies (PWF) in response to 0.07 g (A) and 0.4 g (B) von Frey filament stimuli and paw withdrawal latencies (PWL) to heat stimulation (C) on the contralateral side at the different days as indicated after CCI or sham surgery in mice with pre-microinjection of *Nt3* siRNA or negative control scrambled siRNA (NC siRNA) into unilateral L3/4 DRGs 3 days before CCI or sham surgery. n = 8 mice/group. Three-way ANOVA with repeated measures followed by post hoc Tukey test.

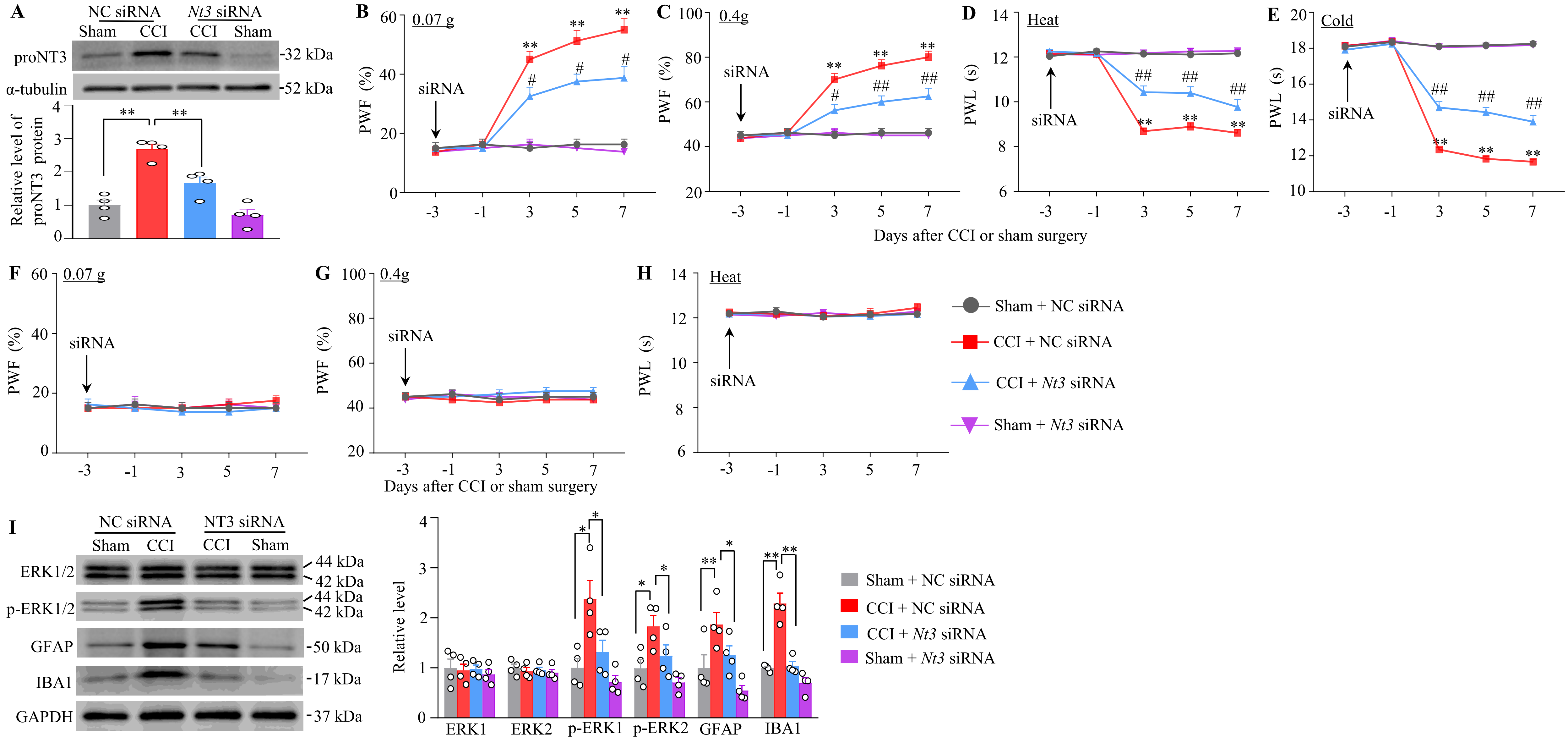
A
NC siRNA
Nt3 siRNA
Sham
CCI
CCI
Sham
proNT3
α-tubulin
32 kDa
52 kDa
Relative level of proNT3 protein
B
0.07 g
siRNA
PWF (%)
C
0.4g
D
Heat
PWL (s)
E
Cold
Days after CCI or sham surgery
F
G
H
Sham + NC siRNA
CCI + NC siRNA
CCI + Nt3 siRNA
Sham + Nt3 siRNA
I
NT3 siRNA
ERK1/2
p-ERK1/2
GFAP
IBA1
GAPDH
44 kDa
42 kDa
50 kDa
17 kDa
37 kDa
Relative level
ERK1
ERK2
p-ERK1
p-ERK2
GFAP
IBA1

Supplementary Figure 3. DRG microinjection of *Nt3* siRNA alleviates the development of nerve trauma-induced nociceptive hypersensitivity in female mice. (A) Expression of proNT3 protein in the ipsilateral L3/4 DRGs on day 7 after CCI or sham surgery in mice with pre-microinjection of *Nt3* siRNA or negative control scrambled siRNA (NC siRNA) into unilateral L3/4 DRGs 3 days before CCI or sham surgery. n = 4 repeats (8 mice)/group. *$P < 0.01$ by two-way ANOVA followed by post hoc Tukey test. (B–H) Paw withdrawal frequencies (PWF) in response to 0.07 g (B and F) and 0.4 g (C and G) von Frey filament stimuli and paw withdrawal latencies (PWL) to heat (D and H) and cold (E) stimuli on the ipsilateral (B-E) and contralateral (F-H) sides at the different days as indicated after CCI or sham surgery in mice with pre-microinjection of *Nt3* siRNA or NC siRNA into unilateral L3/4 DRGs 3 days before CCI or sham surgery. n = 8 mice/group. **$P < 0.01$ versus the NC siRNA plus sham group at the corresponding time points. #$P < 0.05$, ##$P < 0.01$ versus the NC siRNA plus CCI group at the corresponding time points by three-way ANOVA with repeated measures followed by post hoc Tukey test. (I) Expression of total ERK1/2, p-ERK1/2, GFAP and IBA1 proteins in the ipsilateral L3/4 spinal cord dorsal horn on day 7 after CCI or sham surgery in mice with pre-microinjection of *Nt3* siRNA or NC siRNA into unilateral L3/4 DRGs 3 days before CCI or sham surgery. n = 4 repeats (8 mice)/group. *$P < 0.05$, **$P < 0.01$ by two-way ANOVA followed by post hoc Tukey test.

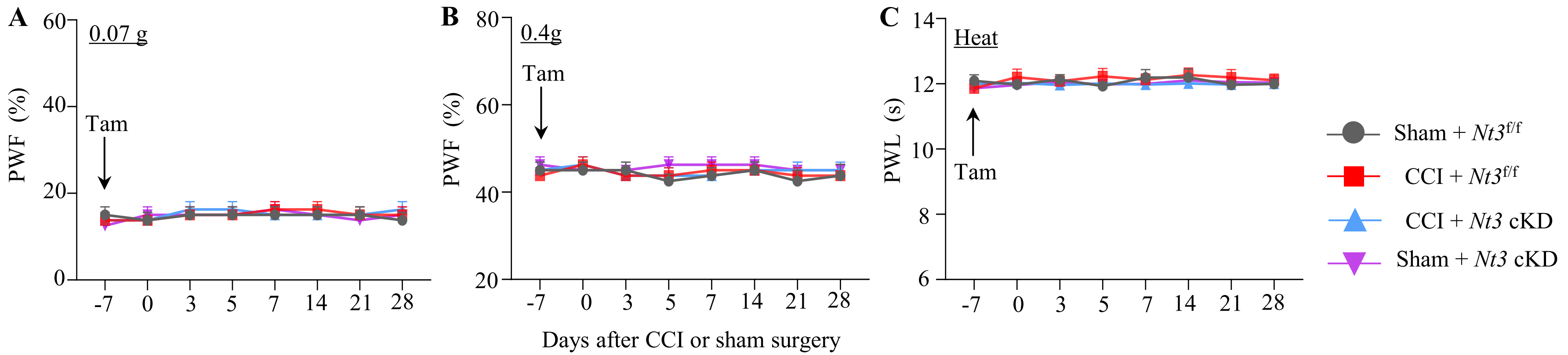

Supplementary Figure 4. Genetic knockdown of *Nt3* gene in DRG sensory neurons does not alter basal behavioral responses on the contralateral side in *Nt3*$^{f/f}$ mice and *Nt3* cKD mice with pre-intraperitoneal injection of tamoxifen (Tam) daily for 7 days. (A-C) Paw withdrawal frequencies (PWF) in response to 0.07 g (A) and 0.4 g (B) von Frey filament stimuli and paw withdrawal latencies (PWL) to heat stimulation (C) on the contralateral sides at the different days as indicated after CCI or sham surgery. n = 8 mice/group. Three-way ANOVA with repeated measures followed by post hoc Tukey test.

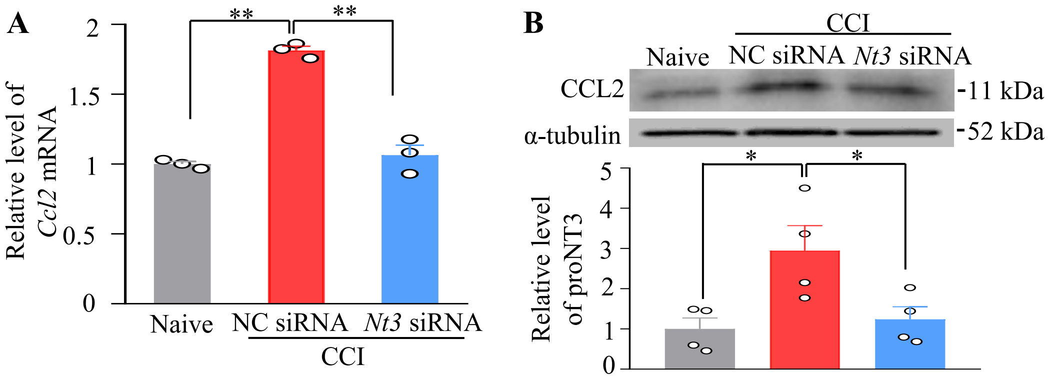

Supplementary Figure 5. Expression of *Ccl2* mRNA (A) and CCL2 protein (B) in the ipsilateral L3/4 DRGs on day 14 after CCI in male mice with post-microinjection of *Nt3* siRNA or negative control scrambled siRNA (NC siRNA) into the ipsilateral L3/4 DRGs 3 days after CCI surgery. n = 3-4 repeats (6-8 mice)/group. **P* < 0.05, ***P* < 0.01 by two-way ANOVA followed by post hoc Tukey test.

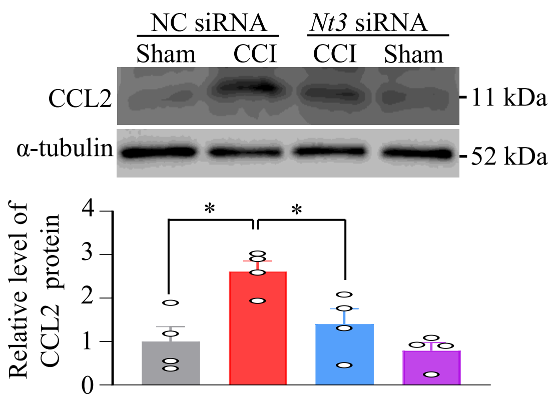

Supplementary Figure 6. Expression of *CCL2* protein in the ipsilateral L3/4 DRGs on day 7 after CCI or sham surgery in female mice with pre-microinjection of *Nt3* siRNA or negative control scrambled siRNA (NC siRNA) into unilateral L3/4 DRGs 3 days before CCI or sham surgery. n = 4 repeats (8 mice)/group. *$P < 0.05$ by two-way ANOVA followed by post hoc Tukey test.

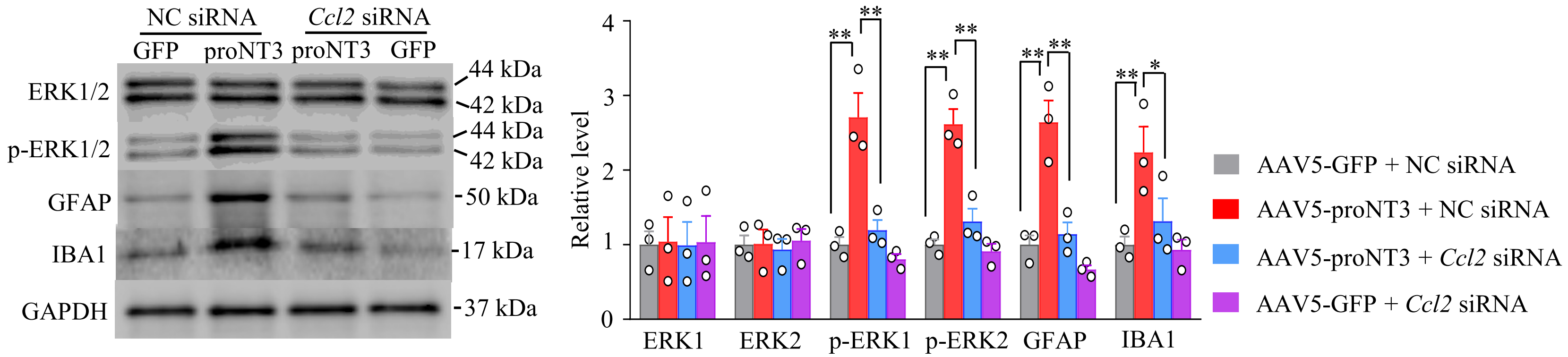

Supplementary Figure 7. DRG *Ccl2* knockdown blocks dorsal horn neuronal and glial hyperactivities caused by DRG proNT3 overexpression. Expression of total ERK1/2, p-ERK1/2, GFAP and IBA1 proteins in the ipsilateral L3/4 spinal cord dorsal horn on day 38 after microinjection of AAV5-proNT3 (proNT3) or AAV5-GFP (GFP) into the unilateral L3/4 DRGs in male mice with post-microinjection of *Ccl2* siRNA or negative control scrambled siRNA (NC siRNA) into the same DRGs 28 days after AAV5 microinjection. n = 3 repeats (6 mice)/group. *$P < 0.05$, **$P < 0.01$ by one-way ANOVA followed by post hoc Tukey test.

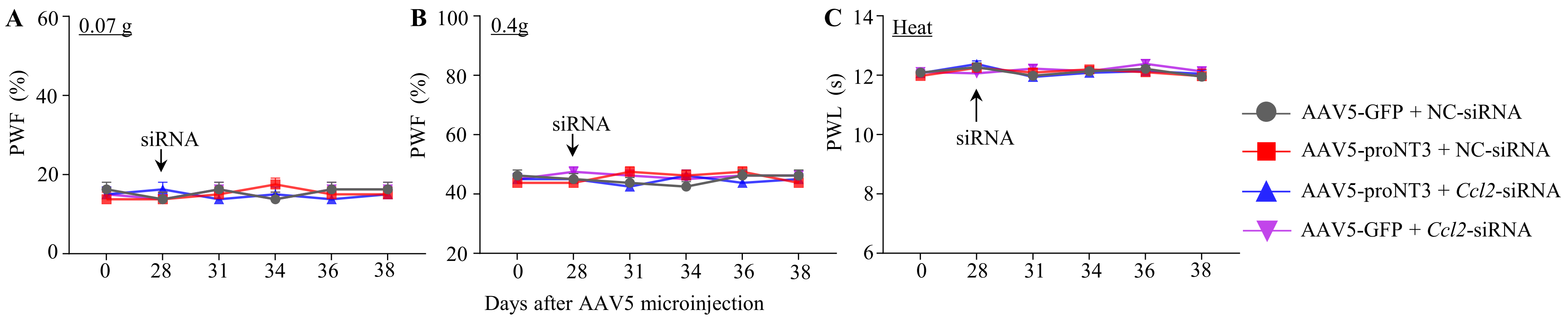

Supplementary Figure 8. DRG microinjection of *Ccl2* siRNA does not alter basal behavioral responses on the contralateral side in male mice. (A-C) Paw withdrawal frequencies (PWF) in response to 0.07 g (A) and 0.4 g (B) von Frey filament stimuli and paw withdrawal latencies (PWL) to heat stimulation (C) on the contralateral sides at the different days as indicated after microinjection of AAV5-proNT3 or AAV5-GFP into the unilateral L3/4 DRGs in male mice with post-microinjection of *Ccl2* siRNA or negative control scrambled siRNA (NC siRNA) into the same DRGs 28 days after AAV5 microinjection. n = 8 mice/group. One-way ANOVA with repeated measures followed by post hoc Tukey test.

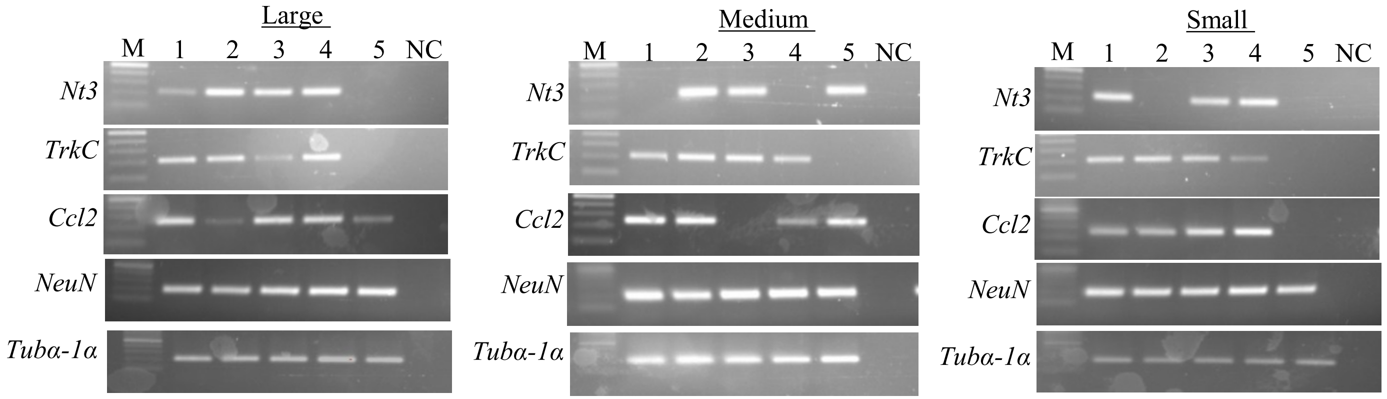

Supplementary Figure 9. Co-expression analysis of *Nt3* mRNA, *TrkC* mRNA and *Ccl2* mRNA in individual large (> 35 µm in diameter), medium (25–35 µm in diameter), and small (< 25 µm in diameter) DRG neurons from naive mice (n = 2) by single-cell RT-PCR assay. *NeuN* mRNA is used as a neuronal marker. *Tuba-1α* mRNA is used as a loading control. M: DNA ladder marker, NC no cDNA. n = 5 neurons/size.

Supplementary Table 1: Primers, probes and siRNAs used

Names	Sequences
**Real-time RT-PCR**	
Tuba1a -F	5′-GTGCATCTCCATCCATGTTG-3′
Tuba1a -R	5′-CAGCTGCTCCACCTTCTTCT-3′
Nt3 -F	5′- ACCACGGAGGAAACGCTATG -3′
Nt3 -R	5′- CCCCGAATGTCAATGGCTGA -3′
Ccl2 -F	5′- CCACTCACCTGCTGCTACTC -3′
Ccl2 -R	5′- GCTGCTGGTGATCCTCTTGT -3′
TrkA -F	5′- TCAAGCGCCAGGACATCATT -3′
TrkA -R	5′- TGGTGCTGTAGCATGGTGAG -3′
TrkB -F	5′- CCTGATGGCCAAGAACGAGT -3′
TrkB -R	5′- GCTTTGGTCAGCAACATCCG-3′
TrkC -F	5′- CATTGCAGAGAACGTGGTGG -3′
TrkC -R	5′- CCCTCCTGGTAGTAGTCCATGT-3′
**Vector construction**	
AAV5- *Nt3* -F	5′- GGCCTCGAGATGTCCATCTTGTTTTATG-3′
AAV5- *Nt3* -R	5′- CGCGCGGCCGCTCATGTTCTTCCAATTTTTC-3′
**Probe**	
Nt3 -F	5′- CGTCTCCTCCTGGATCGTTGGCCACCAGGTCAGAGTTCC -3′
Nt3 -R	5′- GTCTATTCGTATCCAGCGCCA -3′
**siRNAs**	
TrkA	5′- UUCUUGUUGAGAUCACUGGTT-3′
TrkB	5′- CCUGGACAAACUCGUCAGCTT-3′
TrkC	5′- UUGAAGUCCGGUGUAGAGCTC-3′
Ccl2	5′- AUCGGAACCAAAUGAGAUCAG-3′
Nt3	5′- UGUUUCACAGGAGAGUUACTT -3′

F: Forward. R: Reverse.

Supplementary Table 2: Locomotor function

Treatment groups	Placing	Grasping	Righting
Sham + NC siRNA (male)	5 (0)	5 (0)	5 (0)
Sham + NT3 siRNA (male)	5 (0)	5 (0)	5 (0)
CCI + NC siRNA (male)	5 (0)	5 (0)	5 (0)
CCI + NT3 siRNA (male)	5 (0)	5 (0)	5 (0)
$NT3^{f/f}$ + Sham (male)	5 (0)	5 (0)	5 (0)
$NT3^{f/f}$ + CCI (male)	5 (0)	5 (0)	5 (0)
NT3 cKD + Sham (male)	5 (0)	5 (0)	5 (0)
NT3 cKD + CCI (male)	5 (0)	5 (0)	5 (0)
Sham + NC-siRNA (female)	5 (0)	5 (0)	5 (0)
Sham + NT3 siRNA (female)	5 (0)	5 (0)	5 (0)
CCI + NC siRNA (female)	5 (0)	5 (0)	5 (0)
CCI + NT3 siRNA (female)	5 (0)	5 (0)	5 (0)
AAV5-GFP + NC siRNA (male)	5 (0)	5 (0)	5 (0)
AAV5-NT3 + NC siRNA (male)	5 (0)	5 (0)	5 (0)
AAV5-NT3 + TrkA siRNA (male)	5 (0)	5 (0)	5 (0)
AAV5-NT3 + TrkB siRNA (male)	5 (0)	5 (0)	5 (0)
AAV5-NT3 + TrkC siRNA (male)	5 (0)	5 (0)	5 (0)
AAV5-GFP + TrkC siRNA (male)	5 (0)	5 (0)	5 (0)
AAV5-NT3 + CCL2 siRNA (male)	5 (0)	5 (0)	5 (0)
AAV5-GFP + CCL2 siRNA (male)	5 (0)	5 (0)	5 (0)
Sham + Vehicle (male)	5 (0)	5 (0)	5 (0)
Sham + NT3 protein (male)	5 (0)	5 (0)	5 (0)
CCI + Vehicle (male)	5 (0)	5 (0)	5 (0)
CCI + NT3 protein (male)	5 (0)	5 (0)	5 (0)
AAV5-GFP + Vehicle (male)	5 (0)	5 (0)	5 (0)
AAV5-GFP + NT3 protein (male)	5 (0)	5 (0)	5 (0)
AAV5-NT3 + Vehicle (male)	5 (0)	5 (0)	5 (0)
AAV5-NT3 + NT3 protein (male)	5 (0)	5 (0)	5 (0)

n = 8 mice per group; 5 trials; Mean (SEM). GFP: enhanced green fluorescent protein. CCI: chronic constriction injury. NC-siRNA: negative control scramble siRNA. AAV5: recombinant adeno-associated virus types 5. cKD: Sensory neuron-specific inducible conditional NT3 knockdown mice.
